# Integrated Information in Process-Algebraic Compositions

**DOI:** 10.3390/e21080805

**Published:** 2019-08-17

**Authors:** Tommaso Bolognesi

**Affiliations:** Institute of Information Science and Technologies, National Research Council (ISTI-CNR), 1, Via Moruzzi, 56124 Pisa, Italy; t.bolognesi@isti.cnr.it

**Keywords:** boolean net, process algebra, parallel composition operator, integrated information

## Abstract

Integrated Information Theory (IIT) is most typically applied to *Boolean Nets*, a state transition model in which system parts cooperate by *sharing state variables*. By contrast, in *Process Algebra*, whose semantics can also be formulated in terms of (labeled) state transitions, system parts—“processes”—cooperate by *sharing transitions* with matching labels, according to interaction patterns expressed by suitable composition operators. Despite this substantial difference, questioning how much additional information is provided by the integration of the interacting partners above and beyond the sum of their independent contributions appears perfectly legitimate with both types of cooperation. In fact, we collect statistical data about ϕ—integrated information—relative to pairs of boolean nets that cooperate by *three* alternative mechanisms: shared variables—the standard choice for boolean nets—and *two forms* of shared transition, inspired by two process algebras. We name these mechanisms α, β and γ. Quantitative characterizations of all of them are obtained by considering three alternative *execution modes*, namely synchronous, asynchronous and “hybrid”, by exploring the full range of possible coupling degrees in all three cases, and by considering two possible definitions of ϕ based on two alternative notions of distribution distance.

## 1. Introduction

*Integrated Information Theory* (IIT) [1,2] is concerned with the study of natural or artificial systems formed by many interconnected micro-components. One of the key steps in this study is the identification of the “hidden” macro-components of the system, namely its *Minimum Information Partition* (*MIP*). The macro-components of the *MIP* can be seen as distinct but interacting parts: ϕ measures the added value provided by their integration/composition with respect to the plain sum of their contributions—how much the integrated whole is more than the sum of the separate parts.

In *Boolean Nets* [3]—the state transition model predominantly used for illustrating IIT—the integration/cooperation among the *MIP* parts occurs via the directed edges that interconnect them: the parts influence one another by reading each other’s boolean variables—a form of *shared-variable cooperation*.

In this paper, we contrast the above cooperation mechanism with an alternative one based on *shared transitions*, that arises in *Process Algebras* (or “*Calculi*”) [4,5,6,7,8]. Furthermore, viewing the interacting partners under the process algebraic perspective has suggested us to extend our analysis to *three* execution modes for boolean nets: *synchronous*, *asynchronous* and “*hybrid*” (although the second one is soon dropped).

We have three main objectives in mind.

The first is to put the interaction mechanisms of *shared variables* and *shared transitions* on an equal footing, and to obtain some numerical characterization of their “performance” with respect to the ability to produce integrated information.

The second is related to the central application area of IIT—the modeling and quantification of the emergence of consciousness from the complex structure of the brain. Given that the brain architecture is indeed *intrinsically* structured into macro-components, the investigation of alternative or additional mechanisms of cooperation among them, that *explicitly* reflect such higher-level structure, could be an interesting complement to the study of cooperation mechanism that only address micro-components.

The third objective is relevant to the areas from which these additional cooperation mechanisms are borrowed, namely Process Algebra and, more generally, formal methods for Software Engineering. Using informational measures from IIT appears as a completely novel and attractive approach to characterizing quantitatively these practically useful mechanisms and their associated operators.

The paper is organized as follows.

In Section 2, we briefly recall the definition of Boolean Net and introduce the three execution modes: *synchronous*, *asynchronous* and *hybrid*. The first, yielding deterministic behaviors, is the traditional mode; however, the nondeterministic asynchronous and hybrid modes appear more in line with the nondeterminism of the systems typically addressed by Process Algebra. Here, we also discuss the three modes with respect to the property of *conditional independence*.

In Section 3, we introduce the interaction mechanism adopted in process-algebraic calculi/languages, one based on shared labeled transitions (abbreviated “*sharTrans*”) as opposed to shared variables (“*sharVar*”). We in particular illustrate the flexible parametric operator of *parallel composition* from the LOTOS language, denoted “|β|”: expression *P*|β|*Q* describes a system composed of two *processes P* and *Q* that cooperate by sharing some transitions, where β defines the degree of coupling between them.

In Section 4, we show that the parallel composition operator |β| can be readily used also for composing two *boolean nets* *P* and *Q*—still written *P*|β|*Q*—provided these are enriched with transition labels, and regardless of the chosen execution mode. This enables us to put the newly considered form of composition/integration under the lens of IIT without need to import and discuss any other element of Process Algebras.

In Section 5, we introduce notation *P*<α>*Q* and the idea to control the degree of coupling between two bool nets *P* and *Q*, under the *sharVar* mechanism, by controlling the number α of edges crossing between them.

In Section 6, we recall the notion of *integrated information*, the central concept of IIT, both in its *state-dependent form* ϕ(X) and in its *averaged form*, which we denote $\bar{\phi }$. These definitions are based on a distance function $d(y_{coop},y_{indep})$ between two probabilistic state distributions, where $y_{coop}$ reflects inter-part cooperation while $y_{indep}$ corresponds to their independent operation. In IIT 2.0 [1], *d* is *Relative Entropy* (or *Kullback–Liebler divergence*, denoted dkl). We show that the definition of $y_{indep}$ for the *sharVar* context is such to avoid the “*dkl-mismatch problem*” that may arise when applying dkl to generic distributions. Then, we conduct a statistical analysis of ${\bar{\phi }}^{mode}\left(P<\alpha >Q\right)$ in order to study its dependency on α for the sync and hybrid execution modes of *P*<α>*Q*, using 10 pairs (P,Q) of randomly generated bool nets. For facilitating the comparison of *P*<α>*Q* with *sharTrans* compositions (in view of potential dkl-mismatch problems in the latter), we extend our statistical analysis by using a version of $\bar{\phi }$ in which dkl is replaced by *Manhattan distance*.

In Section 7, we address the problem of defining $\bar{\phi }$ in the very different context of *sharTrans* bool net compositions *P*|β|*Q*. Here, we have to face two problems: the presence of deadlocks and the mentioned dkl-mismatches. The first problem is solved easily; a drastic way to bypass the second one is to switch to the $\bar{\phi }$ variant based on Manhattan distance.

Wishing to stick to the original, dkl-based definition of $\bar{\phi }$, in Section 8, we consider an alternative, process-algebraic cooperation mechanism, borrowed from CCS (Calculus of Communicating Systems) [5], that avoids the dkl-mismatch problem. In fact, we combine CCS parallelism (“P|Q”) and restriction (“\γ”) into the convenient syntactic form P[γ]Q≡(P|Q)\γ, where parameter γ still expresses the degree of coupling between the interacting parties. This enables us to compare, by statistical experiments, the trends of $\bar{\phi }$, in its original dkl-based definition [1], for *P*<α>*Q* and P[γ]Q.

In Section 9, we regroup the 15 plots introduced in the previous sections into a compact table that facilitates the comparison of mechanisms α, β and γ.

Some closing remarks are given in Section 10.

## 2. Boolean Nets: Sync, Async and Hybrid Execution Modes

Boolean nets [3] are discrete sequential dynamical systems. An (n,k)-*boolean net* (“bool net” in the sequel) is a pair (G(B,E),F) where:

- G(B,E) is a directed graph with *n* vertices $B=\{b_{1},\ldots ,b_{n}\}$, and edge set *E*; each vertex $b_{i}\in B$ has exactly *k* incoming edges (this limitation on node in-degree is not essential; we adopt it only for convenience of implementation and notation): $b_{i,1}\to b_{i},\ldots ,b_{i,k}\to b_{i}$, so that |E|=nk.
- $F=\{f_{1},\ldots f_{n})$ is a set of *n* boolean functions of *k* arguments, one for each vertex in *B*.

Each vertex $b_{i}\in B$ is a boolean variable controlled by boolean function $f_{i}\left(b_{i,1}\ldots b_{i,k}\right)$ from *F*, where the ordered *k*-tuple of arguments $\left(b_{i,1}\ldots b_{i,k}\right)$ corresponds to the edges incident to $b_{i}$. In the sequel, an (n,k)-bool net *P* is sometimes denoted P(n,k).

A bool net *computation* is a sequence of *steps*, assumed to take place in *discrete time*—one step at each clock tick. Each step consists of the instantaneous and simultaneous firing of a group of nodes, called the *firing group*. A firing group is a subset of *B*, which can be conveniently identified also by its characteristic function (i.e., characteristic function {1,1,0} indicates that only the first two nodes fire, out of three). When node $b_{i}$ fires, its value is updated according to boolean function $f_{i}$.

**Notation.**  Lower case letters *x* and *y* denote discrete *random variables*. In particular, *x* or x(t) is the current state at time *t* of an (n,k)-bool net, consisting of an *n*-tuple of binary random variables $\left(b_{1}\left(t\right)\ldots b_{n}\left(t\right)\right)$. Similarly, *y* or $y\left(t+1\right)=(b_{1}\left(t+1\right)\ldots b_{n}\left(t+1\right))$ is the next state at time (t+1). Upper case letters *X* and *Y* denote actual *n*-tuples of bits, i.e., the values that variables *x* and *y* may assume: $X=(\gamma_{1}\ldots \gamma_{n})$ and $Y=(\delta_{1}\ldots \delta_{n})$, where γs and δs are bits. Subscript *i* in $X_{i}$ is used when we want $X_{i}$ to range in a set of *n*-tuples, for example in the whole set ${\left\{0,1\right\}}^{n}$—*not for selecting an element inside the tuple!* For example, writing $prob(x=X_{i})$, where $X_{i}=\left(\gamma_{1}^{i}\ldots \gamma_{n}^{i}\right)$, means $prob(b_{1}\left(t\right)=\gamma_{1}^{i}\ldots b_{n}\left(t\right)=\gamma_{n}^{i})$. We consistently use identifiers *x* and *X* for predecessor states, and *y* and *Y* for successor states.
  The *densities* of random variables *x* and *y*, often called here “*distributions*”, are denoted $p_{x}$ and $p_{y}$, but sometimes also *x* and *y*, with symbol overloading; the meaning should be clear from the context. For example, the probability for variable *y* to assume value $Y_{i}$ is written $p_{y}\left(Y_{i}\right)$ but also $y(Y_{i})$.
**tpm.**  In the sequel, an essential role is played by the *transition probability matrix* (*tpm*), in which entry $tpm(X_{PQ},Y_{PQ})$ expresses the conditional probability $prob(Y_{PQ}|X_{PQ})$ obtained by counting *all* possible transitions that lead from state $X_{PQ}$ to state $Y_{PQ}$.

Given an (n,k)-bool net, we consider three *execution modes* for it, which differ in the way we define FG, the *set of firing groups* possible at each step. (Note that FG does not depend on the current state.)

**Sync.**  All nodes fire (update) simultaneously. In other words, FG consists of only one firing group, which includes all *n* nodes. For example, when n=3, we have FG={B} (using node sets), also represented as FG={{1,1,1}} (using characteristic functions). Evolution is *deterministic*: each global state has only one successor. *Sync* boolean nets are a generalization of Cellular Automata.
**Async.**  Nodes fire one at a time, the choice being made by a uniform random distribution. In other words, FG consists of *n* firing groups, each being a singleton. For n=3, we have $FG=\{\left\{b_{1}\right\},\left\{b_{2}\right\},\left\{b_{3}\right\}\}$, or FG = {{1,0,0}, {0,1,0}, {0,0,1}}. Evolution is *nondeterministic*: each global state may have multiple successors—as many as *n* (note that, with fixed current state, the correspondence between firing groups and next states can be many-to-one).
**Hybrid.**  (I am thankful to Larissa Albantakis for having drawn my attention to this execution mode and its conditional independence property.) Here, FG consists of $2^{n}$ firing groups—namely, all the subsets of the node set *B*. For n=3, using characteristic functions, we have FG = {{0,0,0}, {0,0,1}, {0,1,0} …{1,1,1}}. The choice is made, again, by a uniform random distribution: the probability to pick any specific firing group is $1/2^{n}$, where *n* is the number of nodes. Note that this is equivalent to firing each node with probability 1/2, independently node by node. Evolution is *nondeterministic*: each global state may have multiple distinct successors—as many as $2^{n}$. Note that the empty firing group is also included.

We write $tpm_{S}^{m}$ for denoting the transition probability matrix of bool net *S* executed in mode *m* (*sync*, *async* or *hybrid*).

We soon deal with composite bool nets. The easiest way to compose two independent bool nets *P* and *Q* is to take their union, defined in the obvious way and denoted P∪Q. *P* and *Q* are disconnected, and do not communicate. It is trivial to see that P∪Q is itself a bool net, which can be executed in any of the three modes.

Let us then establish some simple facts about the relations between the set $FG_{P\cup Q}^{m}$ of firing groups of P∪Q and sets $FG_{P}^{m}$ and $FG_{Q}^{m}$ of firing groups of the components, in the three modes. In the equations below, firing groups are conceived as node sets.

(1) $$FG_{P\cup Q}^{sync}=\left\{B_{P}\cup B_{Q}\right\}=FG_{P}^{sync}\times_{\cup }FG_{Q}^{sync}$$

(2) $$FG_{P\cup Q}^{async}=\left\{\left\{b_{1}\right\}\ldots \left\{b_{p}\right\}\right\}\cup \left\{\left\{b_{1}^{\prime }\right\}\ldots \left\{b_{q}^{\prime }\right\}\right\}=FG_{P}^{async}\cup FG_{Q}^{async}$$

(3) $$FG_{P\cup Q}^{hybrid}=2^{B_{P}\cup B_{Q}}=2^{B_{P}}\times_{\cup }2^{B_{Q}}=FG_{P}^{hybrid}\times_{\cup }FG_{Q}^{hybrid}.$$

In Equation (1), $\left\{B_{P}\cup B_{Q}\right\}$ is a *singleton* set—a set whose unique element is the set $B_{P}\cup B_{Q}$ of nodes. In *sync* mode, FG—be it referred to *P*, *Q* or P∪Q—has only one element, namely the firing group involving all available nodes. Thus, $FG_{P}^{sync}=\left\{B_{P}\right\}$ and $FG_{Q}^{sync}=\left\{B_{Q}\right\}$. Symbol “$\times_{\cup }$” denotes a Cartesian product that takes the union of the paired elements, which are node sets (e.g., $\left\{A,B\right\}\times_{\cup }\left\{C,D\right\}=\left\{A\cup C,A\cup D,\ldots \right\}$.

Executing P∪Q in *async* mode (Equation (2)) means to fire (update) one node at a time. Thus, in this equation, we make use of singleton sets (e.g., $\left\{b_{i}\right\}$ or $\left\{b_{j}^{\prime }\right\}$) formed from the individual nodes of *P* and *Q*, where $B_{P}=\left\{b_{1}\ldots b_{p}\right\}$ and $B_{Q}=\left\{b_{1}^{\prime }\ldots b_{q}^{\prime }\right\}$. Set $FG_{P\cup Q}^{async}$ is then the plain union of sets $FG_{P}^{async}$ and $FG_{Q}^{async}$.

Executing P∪Q in *hybrid* mode (Equation (3)) means to fire any possible subset of $B_{P}\cup B_{Q}$, including the empty set. This set of firing groups can also be seen as the “special” Cartesian product $FG_{P}^{hybrid}\times_{\cup }FG_{Q}^{hybrid}$.

Note that the FG of the whole system is a Cartesian product only for the *sync* and *hybrid* modes, and that these results clearly hold also when *P* and *Q* are connected by some edges, i.e., are not independent, since firing groups are defined relative to node sets, regardless of node interconnections.

### Conditional Independence in the Three Modes

Let $x=\{b_{1}\left(t\right)\ldots b_{n}\left(t\right)\}$ denote the current global state of an (n,k) bool net at time *t*, and $y=\{b_{1}\left(t+1\right)\ldots b_{n}\left(t+1\right)\}$ be the next global state, at time t+1.

Following Pearl [9], we say that, for any i,j∈{1…n}, $b_{i}\left(t+1\right)$ is *conditionally independent from* $b_{j}\left(t+1\right)$, *given x*, if

(4) $$p\left(b_{i}\left(t+1\right)|x,b_{j}\left(t+1\right)\right)=p\left(b_{i}\left(t+1\right)|x\right),\mathrm{when}\;p\left(x,b_{j}\left(t+1\right)\right)>0.$$

Once *x* is known, the additional knowledge of $b_{j}\left(t+1\right)$ does not add anything to what we already know about $b_{i}\left(t+1\right)$ (and vice versa). Note that the above equation means:

(5) $$p\left(b_{i}\left(t+1\right)=\delta_{i}|x=\left(\gamma_{1}\ldots \gamma_{n}\right),b_{j}\left(t+1\right)=\delta_{j}\right)=p\left(b_{i}\left(t+1\right)=\delta_{i}|x=\left(\gamma_{1}\ldots \gamma_{n}\right)\right)$$

for all γs and δs such that $p(x=\left(\gamma_{1}\ldots \gamma_{n}\right),b_{j}\left(t+1\right)=\delta_{j})>0$.

Two random variables $y_{1}$ and $y_{2}$ are *independent* if and only if their *mutual information* [10] is null: $M(y_{1},y_{2})=0$. Similarly, two random variables $y_{1}$ and $y_{2}$ are *conditionally independent*, given *x*, a third variable, if and only if their *conditional mutual information* is null: $M(y_{1},y_{2}|x)=0$.

Recall that *mutual information* $M(y_{1},y_{2})$, a symmetric quantity representing the information provided on average by one variable about the other, is:

(6) $$M\left(y_{1},y_{2}\right)=\sum_{i,j}p_{y_{1}y_{2}}\left(Y_{i},Y_{j}\right)Log_{2}\frac{p_{y_{1}y_{2}}\left(Y_{i},Y_{j}\right)}{p_{y_{1}}\left(Y_{i}\right)p_{y_{2}}\left(Y_{j}\right)},$$

where $p_{y_{1}y_{2}}$ is the joint distribution of the two variables, while $p_{y_{1}}$ and $p_{y_{2}}$ are the respective marginal distributions.

The *conditional mutual information* between variables $y_{1}$ and $y_{2}$, *relative to variable x*, is

(7) $$M\left(y_{1},y_{2}|x\right)=\sum_{i,j,k}p_{y_{1}y_{2}x}\left(Y_{i},Y_{j},X_{k}\right)Log_{2}\frac{p_{y_{1}y_{2}x}\left(Y_{i},Y_{j},X_{k}\right)p_{x}\left(X_{k}\right)}{p_{y_{1}x}\left(Y_{i},X_{k}\right)p_{y_{2}x}\left(Y_{j},X_{k}\right)},$$

which can be also formulated as the weighted sum of the mutual information relative to the individual values $X_{k}$ of variable *x*.

IIT attributes much importance to conditional independence: when the property is satisfied, each element $b_{i}$, with its function $f_{i}\left(b_{i,1}\ldots b_{i,k}\right)$, can be interpreted as an *individual causal element* within the system; when it is violated, a possibly undesirable form of instantaneous causal influence between $b_{i}\left(t+1\right)$ and $b_{j}\left(t+1\right)$ arises.

The three considered execution modes perform differently with respect to conditional independence.

- The *sync* mode entails conditional independence for the simple reason that, due to transition determinism, knowledge of the current state *X* already provides *complete* information about $b_{i}\left(t+1\right)$ (and $b_{j}\left(t+1\right)$).
- With the *async* mode, conditional independence is violated: knowing $b_{j}\left(t+1\right)$, in the case $b_{j}\left(t+1\right)\neq b_{j}\left(t\right)$, reveals that $b_{j}$ has been the only firing (updating) node, which implies $b_{i}\left(t+1\right)=b_{i}\left(t\right)$—a conclusion that we cannot draw from the pure knowledge of *x*.
- The *hybrid* mode entails conditional independence. As already observed, picking a firing group with uniform probability $1/2^{n}$ is equivalent to firing each node with probability 1/2, independently node by node. Thus, finding that $b_{j}$ has fired does not provide additional information on whether or not $b_{i}$ has fired, thus on $b_{i}\left(t+1\right)$.

It is straightforward to see that the above definition of conditional independence, and the results for the three modes, are valid not only for individual nodes but also for groups of nodes, i.e., for parts of the net, such as *P* and *Q* in the sequel.

#### Two-Step Conditional Independence

It could be of some interest to see how conditional dependence/independence carries over to the case of *two or more transitions*, e.g., for analyzing behaviors under macro-transitions, or temporal coarse-graining. Somewhat surprisingly, the scenario changes as follows.

Let x(t), y(t+1), z(t+2) be a sequence of global states; we are now to compare $prob(b_{i}\left(t+2\right)|x\left(t\right),b_{j}\left(t+2\right))$ with $prob(b_{i}\left(t+2\right)|x\left(t\right))$.

In *sync* mode, conditional independence is still valid, for the same argument of the case of one transition: z(t+2) is completely defined, once x(t) is known.

In *async* mode, conditional independence is still violated. If $b_{j}\left(t+2\right)\neq b_{j}\left(t\right)$, we know that node *j* has fired at least once: this fact reduces the probability that node *i* has fired at time t+1 or t+2, providing us additional information about $b_{i}\left(t+2\right)$.

The change occurs with respect to the *hybrid* mode: while the property is satisfied after one transition, it is violated after two. Informally, finding $b_{j}\left(t+2\right)\neq b_{j}\left(t\right)$ reveals that node *j* has fired at least once, which yields additional information about $b_{j}\left(t+1\right)$. This, in turn, may provide additional information about $b_{i}\left(t+2\right)$ beyond what is already given by x(t). Of course, knowing *who fired* in the first step still does not say anything about *who fired* in the second step: the point is that additional knowledge about the intermediate *values* of y(t+1) does refine our knowledge about the possible final *values* of z(t+2).

## 3. Parallel Composition of LOTOS Processes: P|β|Q

In Process Algebras [4,5,6,7,8], a distributed concurrent system is formally described as a set of interacting processes. Each of these formalisms offers its own set of *operators* for specifying actions, interactions, concurrency, choice, nondeterminism, recursion, etc. By the *Structural Operational Semantics* [11], the syntactic expressions built by these operators, describing system structure and behavior, can be formally interpreted as *labeled transition systems*.

Of crucial importance for specifying the macro-structure and interaction patterns of the system are the *parallel composition operators*. We in particular refer to the flexible, parametric parallel composition operator of the process-algebraic language LOTOS (Language of Temporal Ordering Specification) [8].

When two processes *P* and *Q* are composed by the parallel composition operator “|β|”, where β is the set of “synchronization labels”, the resulting labeled transition system is obtained by forcing the processes to proceed jointly—in synchrony—with the transitions with labels in β, while proceeding independently—in “interleaving”—with their other transitions.

The Structural Operational Semantics provides one or more axioms or inference rules specifying the transitions associated with (the expressions formed by) each operator. The inference rules are usually written as “fractions”, and define the transitions of an expression formed by that operator, appearing in the “denominator” (the *conclusion*), in terms of the transitions of the operator arguments, appearing in the “numerator” (the *premise*).

Three inference rules define the semantics of the LOTOS parallel composition expression P|β|Q, where *P* and *Q* are themselves expressions (processes):

(8) $$\frac{P\overset{x}{\to }P^{\prime }\wedge x\notin \beta }{P|\beta |Q\overset{x}{\to }P^{\prime }\left|\beta \right|Q}\;\left(LOTOS\;left\;interleaving\right)$$

(9) $$\frac{Q\overset{x}{\to }Q^{\prime }\wedge x\notin \beta }{P|\beta |Q\overset{x}{\to }P\left|\beta \right|Q^{\prime }}\;\left(LOTOS\;right\;interleaving\right)$$

(10) $$\frac{P\overset{x}{\to }P^{\prime }\wedge Q\overset{x}{\to }Q^{\prime }\wedge x\in \beta }{P|\beta |Q\overset{x}{\to }P^{\prime }\left|\beta \right|Q^{\prime }}\;\left(LOTOS\;synchronization\right)$$

For example, when two processes P[a,b,c] and Q[b,c,d], able to perform transitions with labels in, respectively, sets {a,b,c} and {b,c,d}, are composed by the expression “P[a,b,c]|{b,c}|Q[b,c,d]”, they will interleave their local transitions labeled *a* and *d*, and synchronize those labeled *b* and *c*.

When the set of synchronization labels is empty—β=∅—we have the special case of *pure interleaving* composition P|∅|Q, also denoted P|||Q, where “|||” is called the *interleaving* operator. In this case, it is clear that the rules in Equations (8) and (9) are still applicable while the rule in Equation (10) is not; thus, in composition P|||Q, the components can only proceed one at a time.

In the next section, we discuss how to apply the above parallel composition operator to bool nets, and the way this operator performs with respect to the conditional independence property.

## 4. Parallel Compositions of Bool Nets: P|β|Q

Bool nets are state transition systems, and since the rules in Equations (8)–(10) for parallel composition are applicable to *labeled transition systems*, it is perfectly feasible to apply them to the composition *P*|β|*Q* of boolean nets. The only missing elements are transitions labels!

For our investigations, we adopt pairs (P,Q) of nets with identical (n,k) parameters; for the labels, we proceed as follows.

First, we choose the label *alphabet*, which consists of the set {1,2…2nk} of natural numbers (the choice of size 2nk is justified below). We overload symbol β to denote both a natural number, with 0≤β≤2nk, and the set of *synchronization labels*{1,2…β}, so that P|{1,2…β}|Q is written P|β|Q. In particular, β=0 corresponds to the pure interleaving case P|||Q mentioned in the previous section. As a natural number, β represents the *coupling factor* between *P* and *Q*: the larger is β, the more frequent will be the steps in which *P* and *Q* must synchronize.

Second, we turn *P* and *Q* into *labeled bool nets* by adding two independent functions $L_{P}$ and $L_{Q}$ that, respectively, assign a label to each transition $x_{P}\to y_{P}$ and $x_{Q}\to y_{Q}$:

(11) $$L_{P},L_{Q}:{\left\{0,1\right\}}^{n}\times {\left\{0,1\right\}}^{n}\to \left\{1,2\ldots 2nk\right\}.$$

Aiming at maximum generality, our labels depend both on the source and on the target state, and are picked at random from set {1,2…2nk}.

On this basis, the application of the rules in Equations (8)–(10) to *P*|β|*Q* becomes possible also when *P* and *Q* are labeled bool nets. Note that this can be done regardless of the mode—*sync*, *async* or *hybrid*—in which *P* and *Q* are executed.

It is important not to confuse the concept of *sync*/*async* execution mode of *P* and *Q* with the (orthogonal) concept of *synchronous*/*asynchronous* transition of *P*|β|*Q*. The execution mode refers to the individual component *P* or *Q*, and when we attribute some execution mode to the whole *P*|β|*Q* we mean that both *P* and *Q* operate, *internally*, according to that mode; in principle, we could even imagine composing a *P* operating in *sync* mode with, e.g., a *Q* operating in *async* or *hybrid* mode (but in this paper we never do that). On the other hand, a *synchronous* transition of *P*|β|*Q* is one in which *P* and *Q* proceed jointly, each contributing with a local transition performed according to its own mode; furthermore, the two local and simultaneous transitions must have the same label γ, with γ∈{1,2…β}. Conversely, an *asynchronous* transition of *P*|β|*Q* corresponds to a local, γ-labeled transition performed autonomously (and according to its own mode) by only one of the two components, where γ∉{1,2…β}.

### 4.1. Conditional Dependence in Parallel Composition

We discuss the issue of conditional independence in Section 2.1, relative to pure bool nets. How does parallel bool net composition *P*|β|*Q* perform with respect to this property?

The question involves comparing $prob(y_{P}|x_{PQ},y_{Q})$ with $prob(y_{P}|x_{PQ})$ where, as before, *x* and *y* are states at time *t* and t+1, respectively, and the subscripts identify the relevant system components.

Regardless of the execution mode of the two components, parallel composition *does violate conditional independence*. The reason is that knowing $y_{Q}$ and finding $y_{Q}\neq x_{Q}$ indicates that *Q* has indeed performed a local transition, whose label, e.g., γ, we can partly or completely deduce from labeling function $L_{Q}$, which is known. If γ∉{1,2…β}, the system as a whole must have performed an asynchronous (interleaving) transition, in which *P* must have idled: we immediately deduce $y_{P}=x_{P}$. If, conversely, γ∈{1,2…β}, the system as a whole must have performed a synchronous transition, one in which *P* has performed a γ-labeled transition jointly with *Q*: this still tells us something about $y_{P}$. In both cases, we acquire more information about $y_{P}$ than what $x_{PQ}$ alone can give.

In the area of formal methods for Software Engineering, to which Process Algebras belong, it is indeed conditional *dependence* that plays an important role. Consider, for example, the *constraint-oriented specification style* [12,13]. In this style, the parallel composition operator is used as a sort of logical conjunction: system behavior is specified by progressively accumulating constraints (processes) on the ordering of communication events and, possibly, on the exchanged data values. Each constraint reflects a different, partial view on the global system behavior, and all these views should agree on each global transition x→y. This agreement, governed by the inference rules in Equations (8)–(10), reflects a sort of on-the-fly communication between *P* and *Q*, as the global transition occurs. Overall, the effect of those rules is to introduce a mutual dependency among local transitions, which, in terms of conditional mutual information between local state components, means $M(y_{P},y_{Q}|x_{PQ})\neq 0$.

### 4.2. Deadlocks

No matter which execution mode is considered, a bool net will always be able to perform transitions from any state. This is not the case for bool net *composition* P|β|Q, when β>0. A *deadlock* occurs at global state $X_{PQ}=X_{P}.X_{Q}$, formed by the concatenation of local states $X_{P}$ and $X_{Q}$, when: (i) no *a*-labeled local transitions are possible from state $X_{P}$ *or* $X_{Q}$, with a∉{1…β} (these would become global, interleaving, asynchronous transitions by the rule in Equation (8) or Equation (9)); and (ii) no pair of local *b*-labeled transitions is possible from $X_{P}$ *and* $X_{Q}$, with b∈{1…β} (yielding global, synchronous transitions by the rule in Equation (10)). In this case, $X_{PQ}$ is a *deadlock state*.

Each *tpm* row should be a probability vector: its total must be 1. However, when $X_{PQ}$ is a deadlock state there is no possible successor $Y_{PQ}$, and all elements $tpm_{P|\beta |Q}^{m}\left(X_{PQ},*\right)$ of the corresponding row would be 0s, thus violating the probability vector property. One option sometimes adopted for restoring that property is to set $tpm(X_{PQ},X_{PQ})=1$, forcing the system to permanently remain in that state, and turning a *static* into a *dynamic* deadlock:

**static deadlocks**—some *tpm* rows, called *null rows*, only have 0s, and are not proper probability vectors;
**dynamic deadlocks**—all rows are probability vectors, with loop-edges added.

The introduction of dynamic deadlocks preserves the probabilistic nature of the tpm, but does not discriminate between actual deadlocks and loop-transitions—those for which the source and target state coincide.

Deadlocks tend to increase as the coupling between the interacting parties becomes stronger:

**Proposition** **1.** *Let P and Q be two labeled bool nets, and D(P|β|Q) be the set of deadlock states of system P|β|Q. Then, $\beta_{1}<\beta_{2}$ implies $D(P|\beta_{1}\left|Q\right)\subseteq D(P|\beta_{2}\left|Q\right)$.*

**Proof.**  We prove by contradiction that if global state *x* is a deadlock for $P|\beta_{1}|Q$, it is also a deadlock for $P|\beta_{2}|Q$. Assume *x* is not a deadlock for $P|\beta_{2}|Q$. Then, $P|\beta_{2}|Q$ can perform (at least) a labeled transition $x\overset{a}{\to }y$.

If $a\in \{1\ldots \beta_{2}\}$, the transition is a synchronization between *P* and *Q*, supported by the inference rule in Equation (10): then, either $a\in \{1\ldots \beta_{1}\}$ or $a\in \{\beta_{1}+1\ldots \beta_{2}\}$. In the first case, transition $x\overset{a}{\to }y$ would be feasible also for $P|\beta_{1}|Q$ (a contradiction); in the second case, the two component transitions $x_{P}\overset{a}{\to }y_{P}$ and $x_{Q}\overset{a}{\to }y_{Q}$ would enable, by the inference rules in Equations (8) and (9), two global, interleaving transitions of $P|\beta_{1}|Q$ (a contradiction).

If, on the other hand, $a\notin \{1\ldots \beta_{2}\}$, then $x\overset{a}{\to }y$ is an interleaving transition for $P|\beta_{2}|Q$, which would be a fortiori a feasible interleaving transition for $P|\beta_{1}|Q$ (a contradiction). □

Furthermore, given composition *P*|β|*Q*, we can establish the following relations among the deadlock sets for the different execution modes.

**Proposition** **2.** *Let P and Q be two labeled bool nets, let $P^{mode}\left|\beta \right|Q^{mode}$ be system P|β|Q executed in the specified* mode, *and let D be the deadlock set function of Proposition 1. Then, (i) $D(P^{hybrid}\left|\beta \right|Q^{hybrid})\subseteq D(P^{sync}\left|\beta \right|Q^{sync})$; and (ii) $D(P^{hybrid}\left|\beta \right|Q^{hybrid})\subseteq D(P^{async}\left|\beta \right|Q^{async})$.*

**Proof.**  Part (i). We show by contradiction that, if global state *x* is a deadlock for $P^{hybrid}\left|\beta \right|Q^{hybrid}$, it is also a deadlock for $P^{sync}\left|\beta \right|Q^{sync}$. If *x* were not a deadlock for $P^{sync}\left|\beta \right|Q^{sync}$, then this system could escape state *x* by some transition involving the simultaneous firing of all nodes of $N_{P}$ *and* $N_{Q}$ (by the inference rule in Equation (10)), or the firing of all nodes of $N_{P}$ *or* $N_{Q}$ (by the inference rule in Equation (8) or Equation (9)). These three firing scenarios are feasible also under the *hybrid* execution mode (see the definitions of the firing group sets FG for the three modes in Section 2), yielding a transition escaping state *x* also for system $P^{hybrid}\left|\beta \right|Q^{hybrid}$—a contradiction.

The proof for Part (ii) is analogous. □

**Figure 1:**
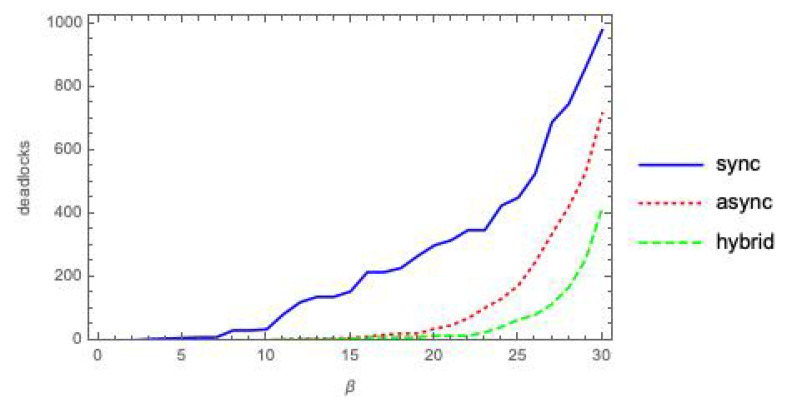
Deadlocks in P(5,3)|β|Q(5,3) as a function of coupling factor β, under *sync*, *async* and *hybrid* execution modes.

Figure 1 shows the count of deadlock states, out of $2^{5+5}=1024$ possible states, as a function of the coupling parameter β, for the parallel composition $P^{m}\left(5,3\right)\left|\beta \right|Q^{m}\left(5,3\right)$ of two randomly generated, labeled (5,3)-bool nets executed in mode *m* = *sync*, *async* or *hybrid*.

The plots in Figure 1 provide experimental evidence for Propositions 1 and 2. Indeed, they might also suggest that a deadlock state *x* for $P^{async}\left|\beta \right|Q^{async}$ must also be a deadlock state for $P^{sync}\left|\beta \right|Q^{sync}$. However, this is not always the case, as shown by the following simple counterexample.

Assume *P* and *Q* are two labeled (n,k)-bool nets with n=2 and k=1. *P* and *Q* have identical topology—node 1 reads node 2 and vice versa—and all nodes are associated with the same *bit-flip* bool function. The label set is {1,2,3,4}, Assume labeling functions $L_{P}$ and $L_{Q}$ are defined so that

- $L_{P}\left(\left(0,0\right),\left(1,1\right)\right)=1,L_{P}\left(\left(0,0\right),\left(1,0\right)\right)=1,L_{P}\left(\left(0,0\right),\left(0,1\right)\right)=2$; and
- $L_{Q}\left(\left(0,0\right),\left(1,1\right)\right)=1,L_{Q}\left(\left(0,0\right),\left(1,0\right)\right)=3,L_{Q}\left(\left(0,0\right),\left(0,1\right)\right)=4$.

Then, if we impose maximum synchronization between *P* and *Q*, by writing P|4|Q, we find that global state x=(0,0,0,0) is a deadlock for $P^{async}\left|4\right|Q^{async}$ (since $P^{async}$ can only perform local transitions $\left(0,0\right)\overset{1}{\to }\left(1,0\right)$ and $\left(0,0\right)\overset{2}{\to }\left(0,1\right)$ while $Q^{async}$ can only offer $\left(0,0\right)\overset{3}{\to }\left(1,0\right)$ and $\left(0,0\right)\overset{4}{\to }\left(0,1\right)$, with no label matching between *P* and *Q*) but it is not a deadlock for $P^{sync}\left|4\right|Q^{sync}$ (since $P^{sync}$ and $Q^{sync}$ can synchronize by performing local transitions $\left(0,0\right)\overset{1}{\to }\left(1,1\right)$).

## 5. Bool Nets as P<α>Q *sharVar* Compositions

In analogy with the expression *P*|β|*Q* for the composition of two separate bool nets *P* and *Q* by shared transitions (Section 4), we let *P*<α>*Q* denote a *single* bool net whose nodes are partitioned into sets $B_{P}$ and $B_{Q}$, and where there are exactly α “*bridges*”, i.e., directed edges with one endpoint in $B_{P}$ and the other in $B_{Q}$. Bridges allow the two bool net parts—called *P* and *Q*—to share and cross-read some of their variables; the other edges are “local” to *P* or *Q*. We take α as the *degree of coupling* between *P* and *Q*. Furthermore, $P^{\alpha }$ and $Q^{\alpha }$, later equivalently denoted P∗ and Q∗, represent the two components *after separation*: a bridge directed from *P* to *Q* (or vice versa) turns into a *dangling edge* of *Q* (or *P*), with no specified source node (the notation $P^{\alpha }$ and $Q^{\alpha }$ is meant to recall the presence of α bridges in the original, uncut bool net; however, it may still happen that one of the components, or both, when α=0, has no dangling edges after separation).

What if we are now given two *independent* bool nets *P* and *Q* and we want to *derive* from them some system *P*<α>*Q* with target coupling factor α? This is done by some surgery: we turn α local edges of *P* and/or *Q* into bridges between *P* and *Q*. The choice of which local edge to turn into a bridge is made at random, and so is the choice of a new source node for it.

Thus, while in building *P*|β|*Q* the two arguments of the composition are unaffected, except for the addition of the labeling functions $L_{P}$ and $L_{Q}$, for building *P*<α>*Q* we do change the topology of the components, although the node sets $B_{P}$ and $B_{Q}$ and the sets of boolean functions $F_{P}$ and $F_{Q}$ are preserved. Strictly, <α> should not be regarded as an algebraic operator, since the operation affects the operands. However, notations *P*<α>*Q* and *P*|β|*Q* are useful for highlighting the system bipartition and the involved degree of coupling.

Let us now clarify a final, subtle point about execution modes for the two types of cooperation—<α> and |β|. While expression $P^{m}\left|\beta \right|Q^{m}$ completely defines the behavior of the system, expression $P^{m}$<α>$Q^{m}$ *would not*.

In the first case, execution mode *m* defines the individual behaviors of $P^{m}$ and $Q^{m}$ in terms of their possible firing groups, while |β| defines the possible transition pairings, i.e., whether or not, given current state $X_{PQ}$, a firing group of *P* can fire simultaneously with one of *Q*, which depends on the involved transition labels. In other words, *m* defines the firing groups at the local level and |β| controls them at the global level, by the mediation of transition labels.

In the second case, the potential firing groups of $P^{m}$ and $Q^{m}$ are well defined too, but we have no indication of how they should be combined to yield global transitions: should they act simultaneously or not? The solution is to understand the execution mode as *applied to the net as a whole.* Correspondingly, the correct, unambiguous notation for the *sharVar* cooperation mechanism would be (P<α>${Q)}^{m}$, although this will often be left implicit.

## 6. Integrated Information $\bar{\phi }$ for P<α>Q (*sharVar*)

In very abstract terms, *state-dependent integrated information* ϕ(Y), relative to a global system state *Y*, reduces to the distance or difference *d* between two *Y*-dependent probabilistic distributions $x_{coop}\left(Y\right)$ and $x_{indep}\left(Y\right)$:

(12) $$\phi \left(Y\right)=d(x_{coop}\left(Y\right),x_{indep}\left(Y\right)).$$

Note the slight abuse of notation: x(Y) denotes here, and in similar contexts in the sequel, a distribution *x* that depends, *as a whole*, on some (state) value *Y*; x(X) elsewhere is used to select a specific element of distribution *x*. The meaning should be clear from the context, and is facilitated by our consistent use of symbols x/X and y/Y, for predecessor and successor states. In IIT terminology, x(Y) denotes a *cause repertoire*, as is clear in Equation (15); similarly, y(X) would denote an *effect repertoire*.

Furthermore, $x_{coop}\left(Y\right)$, $x_{indep}\left(Y\right)$ and consequently ϕ(Y) are defined with a system *partition*{P,Q} in mind (we restrict to bipartitions) (strictly, ϕ should refer to a specific partition, namely the *Minimum Information Partition* (*MIP*) [1,2], but we apply it to any (bi)partition). Distribution $x_{coop}$ refers to the system behavior in which parts *P* and *Q* *cooperate* according to the relevant interaction mechanism, e.g., *sharVar* or *sharTrans*. With distribution $x_{indep}$ the parts are assumed to operate *independently*. Hence, their difference *d* is meant to measure the added value provided by cooperation over independent operation.

In IIT 2.0 [1], *d* is *relative entropy* dkl (Kullback–Leibler divergence):

(13) $$dkl[x_{1}\left|\right|x_{2}]=\sum_{i=1}^{N}x_{1}\left(X_{i}\right)Log_{2}\frac{x_{1}\left(X_{i}\right)}{x_{2}\left(X_{i}\right)},$$

where $x_{1}$ and $x_{2}$ are two distributions on the same discrete domain $\left\{X_{1}\ldots X_{N}\right\}$. Note that dkl[x||x]=0: the dkl of two equal distributions is null. Note also that, in light of Equation (13), one can express the mutual information in Equation (6) as follows:

(14) $$M\left(x_{1},x_{2}\right)=dkl[x_{1}x_{2}\left|\right|x_{1}\times x_{2}],$$

where $x_{1}$ and $x_{2}$ are two random variables with joint distribution denoted $x_{1}x_{2}$ and respective marginal distributions $x_{1}$ and $x_{2}$ (we hope symbol overloading is no too confusing here!). Symbol “×” in Equation (14) denotes distribution product, which is defined in the main text.

Consider an (n,k)-bool net (P<α>${Q)}^{m}$ executed in mode *m* (*sync*, *async* or *hybrid*), denoted PQ for short. The behavior of the net is fully defined by the transition probability matrix $tpm_{PQ}^{m}$. It is easy to see that, regardless of the mode *m*, $tpm_{PQ}^{m}$ cannot have *null-rows* (all 0s), corresponding to deadlocks. Note, however, that one may find *null-columns* with the *sync* and *async* modes, corresponding to “Garden of Eden” states $Y_{PQ}$ that have no predecessor state $X_{PQ}$. This does not happen with the *hybrid* mode, since the firing groups for this mode include the *empty firing group* (no node fires), which creates a loop-edge: any state has itself as a predecessor.

For the subsequent definitions of integrated information for PQ, we also need $tpm_{P*}^{m}$ and $tpm_{Q*}^{m}$: these are the tpms that characterize the *independent* behaviors, under mode *m*, of P∗ and Q∗, i.e., the two components *P* and *Q* after separation, when the data flowing across the α bridges from one to the other are lost due to the cut, and replaced by *white noise*, i.e., uniformly distributed bit tuples.

We are finally ready to actualize the abstract definition of Equation (12) into the concrete definition given in [1]. The state-dependent integrated information $\phi_{P<\alpha >Q}^{m}\left(Y_{PQ}\right)$ for global state $Y_{PQ}$ of (n,k)-bool net PQ = *P*<α>*Q* executed in mode *m* is:

(15) $$\phi_{P<\alpha >Q}^{m}\left(Y_{PQ}\right)=dkl[pre_{P<\alpha >Q}^{m}\left(Y_{PQ}\right)||pre_{P*}^{m}\left(Y_{P}\right)\times pre_{Q*}^{m}\left(Y_{Q}\right)]$$

where:

- $pre_{P<\alpha >Q}^{m}\left(Y_{PQ}\right)$ is the distribution of the *predecessors* of state $Y_{PQ}$, obtained by normalizing $tpm_{P<\alpha >Q}^{m}\left(*,Y_{PQ}\right)$—the $Y_{PQ}$-indexed column of $tpm_{P<\alpha >Q}^{m}$;
- $Y_{P}$ and $Y_{Q}$ are the *P* and *Q* components of state $Y_{PQ}$: $Y_{PQ}=Y_{P}.Y_{Q}$ (concatenation);
- $pre_{P*}^{m}\left(Y_{P}\right)$ and $pre_{Q*}^{m}\left(Y_{Q}\right)$ are the distributions of the predecessors of, respectively, $Y_{P}$ and $Y_{Q}$, obtained as done for $pre_{P<\alpha >Q}^{m}\left(Y_{PQ}\right)$ but using, respectively, $tpm_{P*}^{m}$ and $tpm_{Q*}^{m}$; and
- “×” is distribution multiplication: if $d_{1}$ and $d_{2}$ are probability distributions defined, respectively, over ${\left\{0,1\right\}}^{n1}$ and ${\left\{0,1\right\}}^{n2}$—the sets of tuples of lengths n1 and n2—and $d=d_{1}\times d_{2}$ is the distribution product, then, for the generic (n1+n2)-bit tuple $X^{n1+n2}=X^{n1}.X^{n2}$, we have $d\left(X^{n1+n2}\right)=d\left(X^{n1}\right)*d\left(X^{n2}\right)$.

(The interested reader can find in [14] a freely downloadable demonstration tool illustrating state-dependent ϕ for bool nets executed in the standard, sync mode, for generic partitions.)

The averaged form ${\bar{\phi }}_{dkl}^{m}\left(P<\alpha >Q\right)$ of integrated information (subscript “dkl” is convenient in light of subsequent developments) is defined as a weighted sum over all states $Y_{PQ}$ of the state dependent $\phi_{P<\alpha >Q}^{m}\left(Y_{PQ}\right)$s—a weighted sum that we conveniently express as a dot product (“.”):

(16) $${\bar{\phi }}_{dkl}^{m}\left(P<\alpha >Q\right)=post_{P<\alpha >Q}^{m}\left({\bar{x}}_{PQ}\right).Table\left[\phi_{P<\alpha >Q}^{m}\left(Y_{PQ}\right)|{\left(Y_{PQ}\right)}_{10}=0,1\ldots 2^{n}-1\right]$$

where:

- ${\bar{x}}_{PQ}$ denotes the uniform distribution of PQ states (*n*-tuples of bits);
- $post_{P<\alpha >Q}^{m}\left({\bar{x}}_{PQ}\right)$, expressing the weights of the sum, is the distribution of the *successors* of *state distribution* ${\bar{x}}_{PQ}$. Note that we conceive functions pred and post to be applicable both to a specific state (some bit tuple *X* or *Y*) and to a distribution of such states, e.g., to ${\bar{x}}_{PQ}$. No ambiguity arises, since we always use lowercase to denote random variables or their distributions (*x* and *y*), and uppercase to denote specific state values (*X* and *Y*). (Using a distribution as argument of pred or post is preferred, since a specific state, e.g., state {0,0,1} of a three-bit bool net, can be represented as distribution {0,1,0,0,0,0,0,0} assigning probability 1 to the second triple of bits, when these are presented in lexicographic order, and probability 0 to all other triples. In particular, $post_{PQ}^{m}\left({\bar{x}}_{PQ}\right)={\bar{x}}_{PQ}.tpm_{PQ}$.)
- ${\left(Y_{P}\right)}_{10}$ is the decimal representation of bit tuple $Y_{P}$.
- Table[…] is the list of $\phi_{P<\alpha >Q}^{m}\left(Y_{PQ}\right)$ values for all bit *n*-tuples $Y_{PQ}$, listed in lexicographic order.

In [15], it is shown that ${\bar{\phi }}_{dkl}\left(P<\alpha >Q\right)$ can also be computed as $M\left({\bar{x}}_{PQ},y_{PQ}\right)-\left[M\left({\bar{x}}_{P*},y_{P*}\right)+M\left({\bar{x}}_{Q*},y_{Q*}\right)\right]$, where the barred symbols denote uniform distributions (maximum entropy), and *M* is mutual information between current and next state, both referred to the global system PQ and to the two noised components P∗ and Q∗. In our experiments, we took advantage of this alternative definition, which is computationally more efficient.

### 6.1. The *dkl*-Mismatch Problem for P<α>Q

In light of its definition in Equation (13), $dkl[x_{1}||x_{2}]$ is *undefined* when $x_{1}\left(X_{i}\right)>0$ and $x_{2}\left(X_{i}\right)=0$ for some state $X_{i}$: this is what we call the “dkl-*mismatch problem*”.

Proposition 3 establishes that this problem does not arise with the ${\bar{\phi }}_{dkl}^{m}\left(P<\alpha >Q\right)$ we are considering, at least relative to two execution modes.

**Proposition** **3.** *Given a partitioned bool net P<α>Q, the dkl-mismatch problem does not arise for ${\bar{\phi }}_{dkl}^{m}\left(P<\alpha >Q\right)$, when mode m is* sync *or* hybrid.

**Proof.**  In light of the definition in Equation (15), we must prove that, when an element of distribution $pre_{P<\alpha >Q}^{m}\left(Y_{PQ}\right)$ is different from zero, so is the corresponding element of distribution $pre_{P*}^{m}\left(Y_{P}\right)\times pre_{Q*}^{m}\left(Y_{Q}\right)$. For notational convenience, let these two distributions be called, respectively, $x_{1}$ and $x_{2}$, as in Equation (13). Then, we must prove that, for any state $X_{PQ}$: $x_{1}\left(X_{PQ}\right)>0$ implies $x_{2}\left(X_{PQ}\right)>0$, in *sync* and *hybrid* mode. Now, $x_{1}\left(X_{PQ}\right)>0$ means that there exists (at least) one transition

$$X_{PQ}\overset{fg_{PQ}^{m}}{⟶}Y_{PQ}$$

triggered by some firing group $fg_{PQ}^{m}$. Correspondingly, $tpm_{P<\alpha >Q}^{m}\left(X_{PQ},Y_{PQ}\right)>0$.

Representing firing groups as node sets, and observing that the firing groups of the whole system *P*<α>*Q* and of its parts are independent from α, we can take advantage of Equations (1)–(3), which refer to P∪Q≡*P*<0>*Q*, finding that a global firing group can be decomposed into two local firing groups, under the same mode—$fg_{PQ}^{m}=fg_{P}^{m}\cup fg_{Q}^{m}$—only for m=sync and m=hybrid (for m=async, $fg_{PQ}^{async}$ must include exactly *one* node, while any $fg_{P}^{async}$ and any $fg_{P}^{async}$ must include one node each, so that $fg_{P}^{async}\cup fg_{P}^{async}$ includes *two*).

As a consequence, using a functional notation for transitions, for m=sync and m=hybrid we can write:

$$Y_{PQ}=Y_{P}.Y_{Q}=fg_{PQ}^{m}\left(X_{PQ}\right)=fg_{P}^{m}\left(X_{PQ}\right).fg_{Q}^{m}\left(X_{PQ}\right)=fg_{P}^{m}\left(X_{P}.X_{Q}\right).fg_{Q}^{m}\left(X_{P}.X_{Q}\right).$$

The fact that $Y_{P}=fg_{P}^{m}\left(X_{P}.X_{Q}\right)$ guarantees that $tpm_{P*}^{m}\left(X_{P},Y_{P}\right)>0$: by definition, element $tpm_{P*}^{m}\left(X_{P},Y_{P}\right)$ of “noised” matrix $tpm_{P*}^{m}$ is obtained by the cumulative contribution of all values $tpm_{PQ}^{m}\left(X_{P}.*,Y_{P}.*\right)$, and we have assumed above that at least one of them, namely $tpm_{P<\alpha >Q}^{m}\left(X_{P}.X_{Q},Y_{P}.Y_{Q}\right)$, gives a non-null contribution. Similarly, we find that $tpm_{Q*}^{m}\left(X_{Q},Y_{Q}\right)>0$. We conclude that the cut sub-systems P∗ and Q∗ separately support transitions $X_{P}\to Y_{P}$ and $X_{Q}\to Y_{Q}$, meaning that distribution $pre_{P*}^{m}\left(Y_{P}\right)$ (respectively, $pre_{Q*}^{m}\left(Y_{Q}\right)$) assigns a non-null probability to its $X_{P}$-indexed (respectively, $X_{Q}$-indexed) element. Since $x_{2}$ was defined as $pre_{P*}^{m}\left(Y_{P}\right)\times pre_{Q*}^{m}\left(Y_{Q}\right)$, we conclude that $x_{2}\left(X_{P}.X_{Q}\right)>0$. □

The “anomaly” of the *async* mode already observed in Equations (1)–(3), and in Section 2.1, is further highlighted in Proposition 3. For these reasons, we drop this execution mode, and in the sequel *m* is only *sync* or *hybrid*.

The computation of ${\bar{\phi }}_{dkl}^{m}\left(P<\alpha >Q\right)$ is not even affected by the presence of “Garden of Eden” states. If $Y_{PQ}^{\prime }$ is such a state, we might perhaps represent the predecessor distribution $pre_{P<\alpha >Q}^{m}\left(Y_{PQ}^{\prime }\right)$ as the null “probability vector”; then, regardless of the second argument $pre_{P*}^{m}\left(Y_{P}^{\prime }\right)\times pre_{Q*}^{m}\left(Y_{Q}^{\prime }\right)$ of dkl, we would obtain $\phi_{P<\alpha >Q}^{m}\left(Y_{PQ}^{\prime }\right)=0$. However, in any case, these null values are selected away in the weighted sum of the definition in Equation (16), since state distribution $post_{P<\alpha >Q}^{m}\left({\bar{x}}_{PQ}\right)$—providing the weights—assigns probability 0 to $Y_{PQ}^{\prime }$ since, by the definition of Garden of Eden state, none of the $X_{PQ}$s can transition to the latter.

### 6.2. Statistical Results for ${\bar{\phi }}_{dkl}^{m}\left(P<\alpha >Q\right)$

Having defined ${\bar{\phi }}_{dkl}^{m}\left(P<\alpha >Q\right)$, we wish to investigate the dependence of this measure on α, the degree of coupling between *P* and *Q* when they cooperate by shared variables.

**Figure 2:**
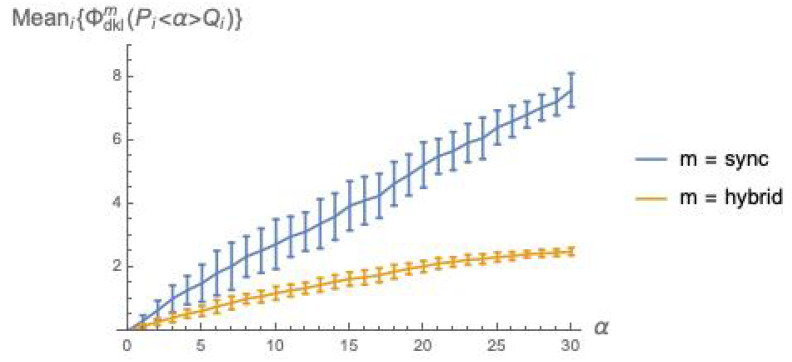
Mean values of ${\bar{\phi }}_{dkl}^{m}(P_{i}$<α>$Q_{i})$, i=1…10, as a function of the coupling factor α, for execution modes sync and hybrid.

Letting n=5 and k=3, we have built ten pairs $\left(P_{i}\left(n,k\right),Q_{i}\left(n,k\right)\right)$, i=1…10, of randomly generated (n,k)-bool nets and have derived, for each pair, the sequence of $P_{i}$<α>$Q_{i}$ systems for values α=0,1…2nk (with 2nk = 30), as described at the beginning of Section 5. Then, for each α, we have computed $Mean_{i=1}^{10}\{{\bar{\phi }}_{dkl}^{m}(P_{i}$<α>$Q_{i})\}$ and associated standard deviation, for m=sync and hybrid. The results of the simulation are shown in the plots of Figure 2.

The plots in Figure 2 confirm the intuitive expectation that integrated information grows with the coupling factor α between *P* and *Q*. Recall that $\bar{\phi }$ is a weighted sum of $\phi (Y_{PQ})s$ (Equation (16)), and that $\phi (Y_{PQ})$ is a dkl “distance” between an $x_{coop}$ and an $x_{indep}$ distribution (Equation (15)). As α grows, it is to be expected that $x_{coop}$ and $x_{indep}$ drift apart, since a larger α means a stronger mutual influence between the behaviors of *P* and *Q*, thus a more marked departure from the behaviors they exhibit when acting independently.

The fact that ${\bar{\phi }}_{dkl}^{sync}>{\bar{\phi }}_{dkl}^{hybrid}$ can be intuitively explained as follows. Due to the different sizes of the involved firing group sets—$|FG_{PQ}^{sync}|=1$ and $|FG_{PQ}^{hybrid}|=2^{2n}$—in sync mode the *successor* distribution $post_{PQ}^{sync}\left(X_{PQ}\right)$ is “punctual” (one element has probability 1, the others have probability 0), while $post_{PQ}^{hybrid}\left(X_{PQ}\right)$ is much more spread over the states. An analogous difference in spread can be observed also when looking backward, with *predecessor* distributions $pre_{PQ}^{sync}\left(Y_{PQ}\right)$ and $pre_{PQ}^{hybrid}\left(Y_{PQ}\right)$. The local *post* and *pre* distributions for *P* and *Q* follow a similar pattern with respect to *sync* vs. *hybrid*. Then, in

$$dkl[pre_{P<\alpha >Q}^{m}\left(Y_{PQ}\right)||pre_{P*}^{m}\left(Y_{P}\right)\times pre_{Q*}^{m}\left(Y_{Q}\right)],$$

the two argument distributions are closer to each other for m=hybrid than for m=sync, due to the higher spread of the distributions for the hybrid mode. Note that the local distributions are also affected by noise injection, which cannot but amplify their spread, pushing them closer to the global distribution with higher spread, namely the distribution for the *hybrid* mode, and smaller distance means smaller $\bar{\phi }$.

### 6.3. Statistical Results for ${\bar{\phi }}_{Manh}^{m}\left(P<\alpha >Q\right)$ Using Manhattan Distance

We show that, for the sharTrans cooperation mechanism |β|, the dkl-mismatch problem becomes pervasive. Thus, for enabling comparisons between sharVar and sharTrans cooperation in terms of integrated information, we consider also a state-dependent version $\hat{\phi }$ of this measure in which the dkl of Equation (15) is replaced by Manhattan distance (*Manh*):

(17) $${\hat{\phi }}_{P<\alpha >Q}^{m}\left(Y_{PQ}\right)=Manh\left(pre_{P<\alpha >Q}^{m}\left(Y_{PQ}\right),pre_{P*}^{m}\left(Y_{P}\right)\times pre_{Q*}^{m}\left(Y_{Q}\right)\right).$$

${\hat{\phi }}_{P<\alpha >Q}^{m}\left(Y_{PQ}\right)$ is then used, as in Equation (16), for obtaining the corresponding state-independent ${\bar{\phi }}_{Manh}^{m}\left(P<\alpha >Q\right)$:

(18) $${\bar{\phi }}_{Manh}^{m}\left(P<\alpha >Q\right)=post_{P<\alpha >Q}^{m}\left({\bar{x}}_{P<\alpha >Q}\right).Table\left[{\hat{\phi }}_{P<\alpha >Q}^{m}\left(Y_{PQ}\right)|{\left(Y_{PQ}\right)}_{10}=0,1\ldots 2^{n}-1\right].$$

**Figure 3:**
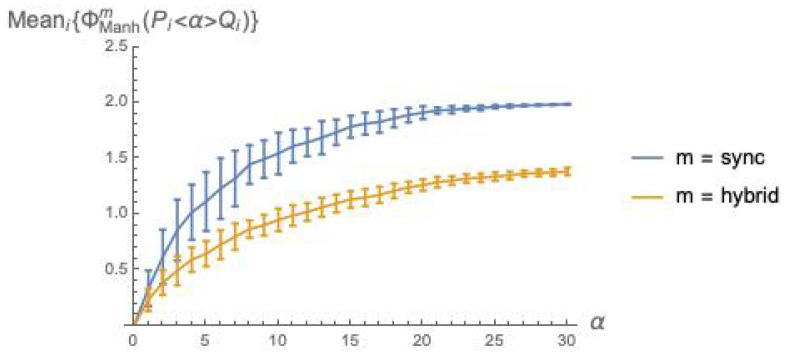
Mean values of ${\bar{\phi }}_{Manh}^{m}(P_{i}$<α>$Q_{i})$, i=1…10, as a function of the coupling factor α, for execution modes sync and hybrid, using *Manhattan distance* in place of dkl.

We have conducted a statistical analysis for ${\bar{\phi }}_{Manh}^{m}\left(P<\alpha >Q\right)$ analogous to that presented in Section 6.2. The results are illustrated in Figure 3. This figure indicates that Manhattan distance broadly agrees with *dkl* (while not suffering from the mismatch problem), and confirms the two general facts already established with Figure 2: integrated information grows with the coupling factor α, and is higher for the *sync* than for the *hybrid* execution mode.

## 7. Integrated Information $\bar{\phi }$ for P|β|Q (LOTOS *sharTrans*)

How can one define integrated information $\bar{\phi }$ for *sharTrans* composition *P*|β|*Q*? The problem reduces to one of adapting to the new context the state-dependent measure ϕ(Y) of Equation (12), which is given a concrete form in Equation (15).

In fact, in $dkl(x_{coop}\left(Y\right),x_{indep}\left(Y\right))$, the first argument is readily defined even in the new setting, since it refers to the “cooperative” behavior of *P*|β|*Q* in which *P* and *Q* interact as specified by operator |β|—a behavior that is fully defined by $tpm_{P|\beta |Q}^{m}$—which is, in turn, fully defined by the inference rules in Equations (8)–(10). The difficulty arises with the definition of $x_{indep}$: what does it mean, in the *sharTrans* context, for *P* and *Q* to *operate independently*?

Our proposed solution to this problem stems from the observation that, while under *sharVar* the cooperating parts share knowledge about each other’s variables, under *sharTrans* they share *knowledge about the order of transitions in time*, since each part must follow the ordering of transitions of the other, at least limited to the transitions whose labels are in the synchronization set β. We must then conclude that the *absence* of cooperation occurs when there is no shared knowledge about local transition ordering, and no concern to agree on it. This immediately suggests to identify independent behavior—and $x_{indep}$ in expression $dkl(x_{coop},x_{indep})$—with the pure interleaving composition P|||Q (see Section 3). The resulting definition of state-dependent integrated information for the |β| mechanism is then:

(19) $$\phi_{P|\beta |Q}^{m}\left(Y_{PQ}\right)=dkl[pre_{P|\beta |Q}^{m}\left(Y_{PQ}\right)||pre_{P|||Q}^{m}\left(Y_{PQ}\right)].$$

Note that the above definition is conceptually (and computationally) simpler than the corresponding definition for <α> (Equation (15)): no input noise for the cut components (P∗ and Q∗) is involved, and the second argument of dkl is not a distribution product but simply the first element of the sequence *P*|β|*Q* for β=0,1….

Then, the definitions of *state-independent* integrated information $\bar{\phi }$ for *P*|β|*Q* and for *P*<α>*Q* are essentially the same (compare with Equation (16)):

(20) $${\bar{\phi }}_{dkl}^{m}\left(P|\beta |Q\right)=post_{P|\beta |Q}^{m}\left({\bar{x}}_{PQ}\right).Table\left[\phi_{P|\beta |Q}^{m}\left(Y_{PQ}\right)|{\left(Y_{PQ}\right)}_{10}=0,1\ldots 2^{2n}-1\right].$$

Note that *n* is now the number of nodes in *P*, which has the same size as *Q*, yielding a total of 2n nodes.

### 7.1. The *dkl*-Mismatch Problem for P|β|Q

As anticipated, the dkl-mismatch problem becomes pervasive with systems of type *P*|β|*Q*. Let state $Y_{PQ}$ be fixed and consider distributions $x_{1}=pre_{P|\beta |Q}^{m}\left(Y_{PQ}\right)$ and $x_{2}=pre_{P|||Q}^{m}\left(Y_{PQ}\right)$ in Equation (19). The mismatch occurs when $x_{1}\left(X_{PQ}^{\prime }\right)>0$ and $x_{2}\left(X_{PQ}^{\prime }\right)=0$ for some $X_{PQ}^{\prime }$. In *sync* mode, it is easy to imagine a transition $X_{PQ}^{\prime }\overset{P|\beta |Q}{⟶}Y_{PQ}$ of system *P*|β|*Q* such that the two bit tuples $X_{PQ}^{\prime }$ and $Y_{PQ}$ differ both in their *P* and in their *Q* component: this may happen when the transition is a synchronization. Since $X_{PQ}^{\prime }$ is a predecessor state of $Y_{PQ}$, we have $x_{1}\left(X_{PQ}^{\prime }\right)>0$. On the contrary, no predecessor of $Y_{PQ}$ under system P|||Q can differ from $Y_{PQ}$ in both state components, since system P|||Q must fire one component at a time, as explained at the end of Section 3. Thus, necessarily, $x_{2}\left(X_{PQ}^{\prime }\right)=0$: this yields the mismatch. The argument for the hybrid mode is analogous, the key point being that a firing group of *P*|β|*Q* may involve nodes from both *P* and *Q*, while a firing group of P|||Q involves nodes exclusively from one component, by the definition of “|||”.

**Figure 4:**
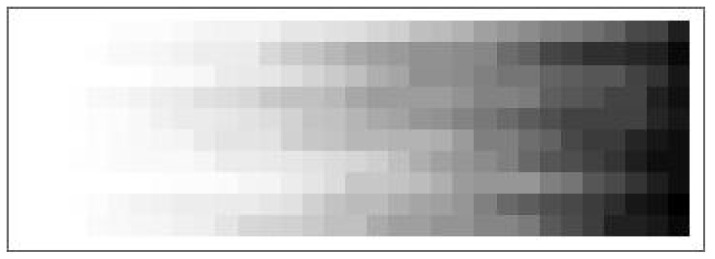
Number of states, represented as grey levels, that give rise to a dkl-mismatch for systems $P_{i}^{sync}$<β>$Q_{i}^{sync}$, i=1…10 (rows), β=0…30 (columns). Systems have up to 993 states, out of 1024, that yield mismatch (the darkest cells), and only 16 systems, out of 310, have none.

To give an idea of how severe the dkl-mismatch problem is for *P*|β|*Q*, we have counted the number of states $Y_{PQ}$ yielding a dkl-mismatch for each of the 310 systems $(P_{i}$<β>$Q_{i})$, for i=1…10 and β=0…30, where the $\left(P_{i},Q_{i}\right)$ pairs are those already used in Section 6 (Figure 2). These numbers are collected in the 10×31 grey-level matrix of Figure 4.

### 7.2. Statistical Results for ${\bar{\phi }}_{Manh}^{m}\left(P|\beta |Q\right)$ Using Manhattan Distance

In light of the impossibility to use the dkl-based definition of ϕ (Equation (19)) for |β| cooperation, we switch, again, to a “hat” version $\hat{\phi }$ in which dkl is replaced by *Manhattan distance*:

(21) $${\hat{\phi }}_{P|\beta |Q}^{m}\left(Y_{PQ}\right)=Manh\left(pre_{P|\beta |Q}^{m}\left(Y_{PQ}\right),pre_{P|||Q}^{m}\left(Y_{PQ}\right)\right),$$

and use it, in turn, for defining the state-independent ${\bar{\phi }}_{Manh}^{m}\left(P|\beta |Q\right)$, as in Equation (20):

(22) $${\bar{\phi }}_{Manh}^{m}\left(P|\beta |Q\right)=post_{P|\beta |Q}^{m}\left({\bar{x}}_{PQ}\right).Table\left[{\hat{\phi }}_{P|\beta |Q}^{m}\left(Y_{PQ}\right)|{\left(Y_{PQ}\right)}_{10}=0,1\ldots 2^{2n}-1\right].$$

**Figure 5:**
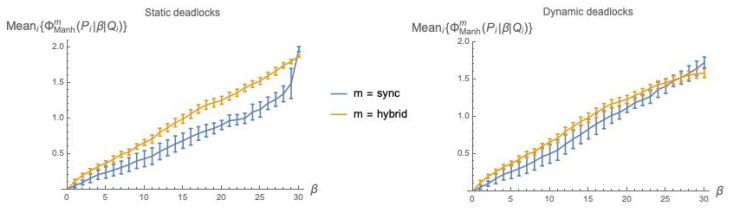
Mean values of ${\bar{\phi }}_{Manh}^{m}(P_{i}\left|\beta \right|Q_{i})$, i=1…10, as a function of the coupling factor β, for execution modes sync and hybrid, using *Manhattan distance* in place of dkl, using: static deadlocks (**left**); and dynamic deadlocks (**right**).

Analogous to Figure 3, in Figure 5, we plot the values of ${\bar{\phi }}_{Manh}^{m}\left(P|\beta |Q\right)$ as a function of the coupling factor β, each point obtained by averaging over 10 (P,Q) pairs, both using static deadlocks (Figure 5, left) and dynamic deadlocks (Figure 5, right).

The distinction between static and dynamic deadlocks was introduced in Section 4.2. Note that when using static deadlocks—no 1s added on the diagonal of $tpm_{P|\beta |Q}^{m}$—the weights $post_{P|\beta |Q}^{m}\left({\bar{x}}_{PQ}\right)$ in Equation (22) will in general not total 1, *and must be re-normalized*.

## 8. Integrated Information $\bar{\phi }$ for P[γ]Q (CCS *sharTrans*)

Kullback–Leibler divergence dkl is a central element of Integrated Information Theory 2.0 [1], thua it is indeed desirable to apply it in the new *sharTrans* context without incurring the dkl-mismatch problem. In this section, we propose a slightly different version of *sharTrans* cooperation, directly inspired to Robin Milner’s seminal process algebra CCS (Calculus of Communicating Systems) [5], that precisely avoids that problem.

Consider the abstract expression:

$$dkl[pre_{coop}^{m}\left(Y_{PQ}\right)||pre_{indep}^{m}\left(Y_{PQ}\right)].$$

How can we conceive the cooperative PcoopQ and independent PindepQ behaviors of bipartite system PQ (as defined by matrices $tpm_{coop}^{m}$ and $tpm_{indep}^{m}$, from which $pre_{coop}^{m}\left(Y_{PQ}\right)$ and $pre_{indep}^{m}\left(Y_{PQ}\right)$ are derived) so that the dkl-mismatch problem is ruled out?

Clearly, a *sufficient condition* for avoiding dkl-mismatches is the following:

The existence of a transition $X\overset{PcoopQ}{⟶}Y$ *implies* the existence of transition $X\overset{PindepQ}{⟶}Y$ between the same states.

The two CCS behavioral operators of (non-parametric) *parallel composition* (“P|Q”) and (parametric) *restriction* (“\γ”), where γ is a label set {1,2…γ} (using the same convention as for β), offer us a way to define cooperation and independence so that they satisfy the above condition.

In CCS, symbol τ denotes a special *internal*, *not observable* transition label: no synchronization is possible with a process that performs a τ-labeled transition. Let *A* be the set of observable labels and define $A^{+}=A\cup \left\{\tau \right\}$. Symbol *a* ranges in *A* and symbol *x* ranges in $A^{+}$. We provide below the four inference rules of the Structural Operational Semantics of CCS for the two mentioned operators (we depart from the standard definition of Milner [5] only in one aspect: we drop the idea of a synchronization based on the matching between a label *a* and its corresponding “co-label” $\bar{a}$, and revert to the LOTOS requirement that the two labels be simply equal).

(23) $$\frac{P\overset{x}{\to }P^{\prime }\wedge x\in A^{+}}{P|Q\overset{x}{\to }P^{\prime }|Q}\;\left(CCS\;left\;interleaving\right)$$

(24) $$\frac{Q\overset{x}{\to }Q^{\prime }\wedge x\in A^{+}}{P|Q\overset{x}{\to }P|Q^{\prime }}\;\left(CCS\;right\;interleaving\right)$$

(25) $$\frac{P\overset{a}{\to }P^{\prime }\wedge Q\overset{a}{\to }Q^{\prime }\wedge a\in A}{P|Q\overset{\tau }{\to }P^{\prime }|Q^{\prime }}\;\left(CCS\;synchronization\right)$$

(26) $$\frac{P\overset{a}{\to }P^{\prime }\wedge a\notin \gamma }{P\backslash \gamma \overset{a}{\to }P^{\prime }}\;\left(CCS\;restriction\right)$$

The “interleaving” rules in Equations (23) and (24) establish that any transition that *P* or *Q* could perform *locally*—in itself—can be performed *globally* by composite system P|Q. Additionally, the rule in Equation (25) establishes that parallel composition P|Q can also perform synchronization transitions whenever two equally labeled observable transitions are available at the two sides. The rule in Equation (26) defines the restriction P\γ as a filter that enables *P* (which can be itself a two-process composition) to perform a transition only if its label is not in the specified set γ of *forbidden* labels (thus, τ is always admitted), pruning away all other transitions.

We now combine CCS *parallel composition* and *restriction* into the convenient syntactic form

(27) P[γ]Q≡(P|Q)\γ,

where parameter γ is enclosed in square brackets to distinguish it from the LOTOS form “|β|”, and use it for actualizing the cooperation and independence relations between *P* and *Q*:

(28) $$\begin{array}{ccc} PindepQ & = & P|Q\equiv P[0]Q \end{array}$$

(29) $$\begin{array}{ccc} PcoopQ & = & P[\gamma ]Q. \end{array}$$

Note that no deadlock can occur in P|Q. With the LOTOS-based *sharTrans* composition, we had assumed PindepQ=P|||Q, a form of independence by which *P* and *Q* will never deadlock *and* will *never* synchronize: their respective transitions can only interleave. This is not the case for the CCS-based approach, where PindepQ=P[0]Q=P|Q: the two independent systems, by the rule in Equation (25), can indeed synchronize any pair of transitions $P\overset{a}{\to }P^{\prime }$ and $Q\overset{a}{\to }Q^{\prime }$ with the same observable label. However, by the rules in Equations (23) and (24), these same transitions can be executed separately, in interleaving—in *independence*. Cooperation P[γ]Q≡(P|Q)\γ, then, consists in ruling out these independent transitions—at least those specified in set γ—while preserving their synchronizations.

One could argue that P|Q already entails a sort of cooperation, via all the synchronization transitions it supports, and that pure LOTOS interleaving P|||Q is a more appropriate form of independence. This is true only in part. The “cooperation” that takes place in P|Q is *not private*: the transition $P\overset{a}{\to }P^{\prime }$ that *P* shares with *Q*, forming a τ-labeled synchronization, is also “offered” separately by *P* (and by *Q* too) for further two-way synchronizations with other potential partners. When we apply restriction to the composition—(P|Q)\γ—we rule out this possibility, and cooperation via joint transitions with labels in γ becomes exclusive of the (P,Q) pair, occurring via a global, τ-labeled transition. In other words, in P|Q, the parties are not *forced* to wait for each other at specific transitions, as in *P*|β|*Q*, while this effect of mutual influence on transition ordering is enforced in P[γ]Q, when restriction is in action.

It is clear that the above sufficient condition for ruling out dkl-mismatches is satisfied by our newly adopted CCS-based definitions:

(30) $$\begin{array}{ccc} \phi_{P[\gamma ]Q}^{m} & = & dkl[pre_{P[\gamma ]Q}^{m}\left(Y_{PQ}\right)||pre_{P[0]Q}^{m}\left(Y_{PQ}\right)] \end{array}$$

(31) $$\begin{array}{ccc} {\bar{\phi }}_{dkl}^{m}\left(P\left[\gamma \right]Q\right) & = & post_{P[\gamma ]Q}^{m}\left({\bar{x}}_{PQ}\right).Table\left[\phi_{P[\gamma ]Q}^{m}\left(Y_{PQ}\right)|{\left(Y_{PQ}\right)}_{10}=0,1\ldots 2^{2n}-1\right] \end{array}$$

since, by the definition of the restriction operator, the transitions of P[γ]Q are a subset of those of P[0]Q.

### 8.1. Deadlocks in P[γ]Q

While deadlocks can never occur in P|Q, they may occur in P[γ]Q≡(P|Q)\γ: this happens when *P* and *Q* offer disjoint sets of labels, thus preventing any synchronization between them, and when all these labels are members of γ, the set of forbidden labels.

**Figure 6:**
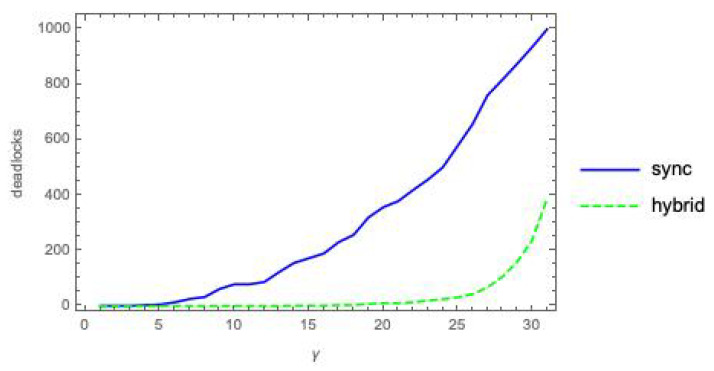
Deadlocks in P(5,3)|γ|Q(5,3) as a function of coupling factor γ, under *sync* and *hybrid* execution modes.

In Figure 6, we show the count of deadlock states, out of 1024 possible states, as a function of the coupling parameter γ, for the composition $P^{m}\left(5,3\right)\left[\gamma \right]Q^{m}\left(5,3\right)$ of two randomly generated labeled (5,3)-bool nets executed in modes *sync* and *hybrid*.

Recall that we have dropped the async mode for its various anomalies. For the remaining two modes, deadlocks under the [γ] and |β| interaction mechanisms seem to behave quite similarly (compare with Figure 1).

### 8.2. Statistical Results for ${\bar{\phi }}_{dkl}^{m}\left(P\left[\gamma \right]Q\right)$

Before presenting the plots, we need to deal again with static vs. dynamic deadlocks (Section 4.2). Referring to the *sync* mode, tpms with dynamic deadlocks turn out to be inappropriate for computing ${\bar{\phi }}_{dkl}^{sync}\left(P\left[\gamma \right]Q\right)$, since they would re-introduce the dkl-mismatch problem that we have managed to rule out by switching to the [γ] cooperation mechanism! The reason is that a static deadlock is made dynamic by adding a “1” on the diagonal of $tpm_{P[\gamma ]Q}^{sync}$, at an otherwise null row. This entry is unlikely to find a non-zero counterpart in $tpm_{P[0]Q}^{sync}$, since P[0]Q (i.e., P|Q) has no deadlocks—no 1s added on the diagonal—and the only possibility to have a non-zero entry on the diagonal is that an actual loop-transition $X_{PQ}\to X_{PQ}$ be possible for that system. However, when *P* and *Q* are executed in *sync* mode, this is unlikely, both when they operate in interleaving (i.e., when only one of them updates all its nodes) and, even worse, when they synchronize (i.e., when all nodes of *P* and *Q* are updated). Thus, for the sync mode, the option is to use static deadlocks—no 1s added on the diagonal of $tpm_{P[\gamma ]Q}^{m}$. As observed above for the |β| composition, the weights $post_{P[\gamma ]Q}^{m}\left({\bar{x}}_{PQ}\right)$ in Equation (22), will in general not total 1, and must be re-normalized.

The dkl-mismatch problem does not arise with the *hybrid* mode since, using the empty firing group, a loop edge $X_{PQ}\to X_{PQ}$ is always possible for system P|Q, for any state $X_{PQ}$, so the elements on the diagonal of $tpm_{P[0]Q}^{hybrid}$ are all different from 0. In this case, we can then safely use both dynamic deadlocks and static deadlocks with re-normalization.

**Figure 7:**
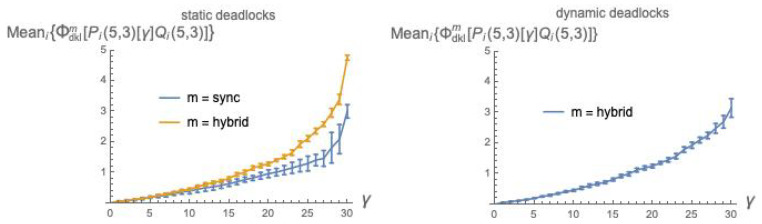
Mean values of ${\bar{\phi }}_{dkl}^{m}\left(P_{i}\left[\gamma \right]Q_{i}\right)$, i=1…10, as a function of the coupling factor γ, for execution modes sync and hybrid, using the definitions in Equations (30) and (31). The used tpms implement: static deadlocks (**left**); and dynamic deadlocks (**right**).

In Figure 7, we plot the values of ${\bar{\phi }}_{dkl}^{m}\left(P\left[\gamma \right]Q\right)$ as a function of the coupling factor γ, each point obtained by averaging over 10 (P,Q) pairs, using static deadlocks (for the *sync* and *hybrid* modes) and dynamic deadlocks (only for the *hybrid* mode).

### 8.3. Statistical Results for ${\bar{\phi }}_{Manh}^{m}\left(P\left[\gamma \right]Q\right)$ Using Manhattan Distance

The potential mismatch between distributions $d_{1}$ and $d_{2}$ in $dkl[d_{1}||d_{2}]$ is not a concern when using Manhattan distance $Manh(d_{1},d_{2})$ for defining ${\bar{\phi }}_{Manh}^{m}\left(P\left[\gamma \right]Q\right)$ (by equations analogous to Equations (30) and (31)). Thus, we can handle both static deadlocks, with renormalization of the weights, and dynamic deadlocks.

**Figure 8:**
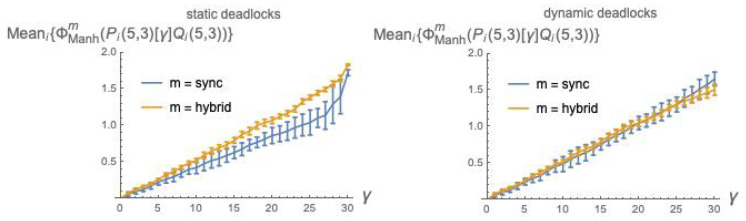
Mean values of ${\bar{\phi }}_{Manh}^{m}\left(P_{i}\left[\gamma \right]Q_{i}\right)$, i=1…10, as a function of the coupling factor γ, for execution modes sync and hybrid, using: static deadlocks (**left**); and dynamic deadlocks (**right**).

Figure 8 is analogous to Figure 7, except that Manhattan distance is used in place of dkl.

## 9. Comparisons

Our experimental analysis has involved three dimensions, or degrees of freedom:

- (i) bool net cooperation mechanism (α/β/γ);
- (ii) bool net execution mode (sync/hybrid); and
- (iii) “distance” function for probabilistic distributions (dkl/*Manhattan distance*).

**Figure 9:**
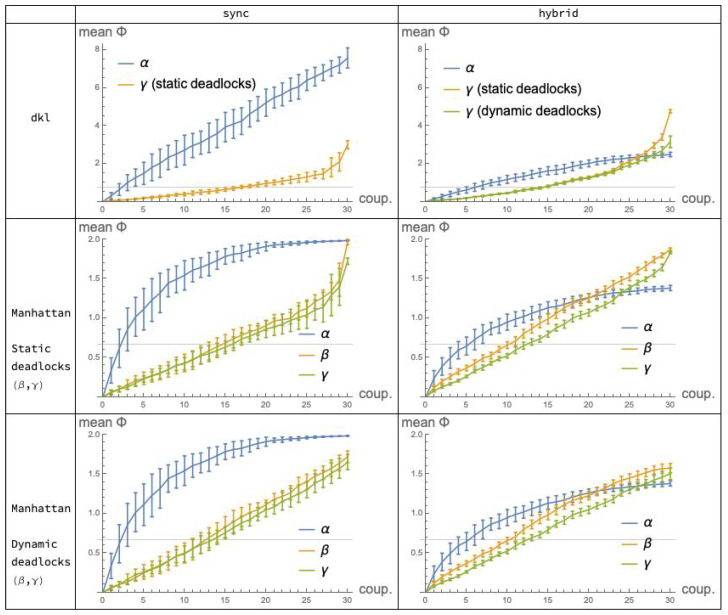
Rearranging the plots of the previous sections for a comparison of the α, β, γ cooperation mechanisms, given a particular choice of execution mode (**columns**) and distance function (**rows**). For Manhattan distance, we differentiate between systems with static or dynamic deadlocks (**Rows 2 and 3**).

As stated initially, our interest is primarily in the comparison of cooperation mechanisms (i). It is then useful to aggregate the statistical data collected in the previous sections so that the plots for α, β and γ appear in the same diagram. This is done in Figure 9 which shows, for each fixed choice of execution mode (the columns) and distance function (the rows), the “performance” of the applicable mechanisms in terms of integrated information, as a function of the coupling factor (“coup”). Note that Manhattan distance is represented in Rows 2 *and* 3, corresponding to systems implementing, respectively, static and dynamic deadlocks: this distinction only affects the β and γ plots, since the α mechanism is immune to deadlocks. For convenience, the α plots in Row 2 are replicated in Row 3. (Recall also that the *dkl*-mismatch problem prevented us from applying distance dkl to the β mechanism.)

In the following subsections, we consider all three “dimensions” (iii), (ii) and (i), precisely in this order, with (i) being the dominant one.

### 9.1. Distribution Distances: Kullback–Leibler Divergence *dkl* vs. Manhattan

It is not our goal here to assess the various (pseudo-)distances used for defining $\bar{\phi }$: the interested reader can find an accurate study involving *seven* options for this metric in [16]. However, we are interested in checking whether, despite the different ranges of values and plot shapes that they yield, the two alternative distances give analogous indications about the *mutual relations* among the α, β and γ mechanisms.

A quick look at the grid of Figure 9 suggests that the relations between the plots for α and γ are not “qualitatively” different under *dkl* (Row 1) and under Manhattan distance (Rows 2 and 3). By this, we mean that the *relative order* between $\bar{\phi }$ values for the different mechanisms and modes, as the coupling factor moves in its range, is substantially the same for the two distances.

More precisely, we can split the comparisons in two steps.

*Fix the mode and vary the mechanism.* In mode sync (Column 1 of Figure 9), the relation between the α and γ plots is qualitatively the same for dkl and for *Manhattan distance*. A similar observation applies relative to mode hybrid (Column 2).

*Fix the mechanism and vary the mode*. Consider the α mechanism: under dkl, the relation between ${\bar{\phi }}_{dkl}^{sync}$ and ${\bar{\phi }}_{dkl}^{hybrid}$ is depicted in Figure 2; under Manhattan distance, the relation between ${\bar{\phi }}_{manh}^{sync}$ and ${\bar{\phi }}_{manh}^{hybrid}$ is qualitatively the same, and is depicted in Figure 3. For mechanism β, the comparison dkl/*Manhattan distance* does not apply. For mechanism γ, under dkl the relation between ${\bar{\phi }}_{dkl}^{sync}$ and ${\bar{\phi }}_{dkl}^{hybrid}$ is depicted in Figure 7 (left), where static deadlocks are assumed. An analogous relationship is observed between ${\bar{\phi }}_{manh}^{sync}$ and ${\bar{\phi }}_{manh}^{hybrid}$ in Figure 8 (left) that also assumes static deadlocks.

In conclusion, the choice to use *Manhattan distance* as an alternative to dkl, for comparing the α, β and γ mechanisms appears, a posteriori, convenient and fully legitimate.

### 9.2. Modes: Sync vs. Hybrid

Having found that the choice of distribution distance does not affect the picture of the relations among α, β and γ, we may wonder whether the same happens with the choice between *sync* and *hybrid* mode. It turns out that this is *not* the case: the pictures that emerge under the two modes are different, as a quick comparison of the two columns of Figure 9 reveals. In light of the findings of the previous subsection, it is sufficient, convenient and safe to focus on Manhattan distance—Tows 2 and 3. Indeed, given the minimal differences between these two rows, choosing one or the other is irrelevant: implementing static or dynamic deadlocks in the tpms does not significantly affect our comparisons.

In *sync* mode, the α plot is constantly higher than the β and γ plots, which are close to each other; in *hybrid* mode, the α plot crosses the other two. This crossing is mainly due to a substantial *decrement* of the values of the α plots, when switching from sync to hybrid (Figure 3)—a justification of this is given in Section 6.2—whereas for the β mechanism the effect of the mode switch is reversed, with a moderate *increase* of the values of the *hybrid* over the *sync* plot (Figure 5). For the γ mechanisms (Figure 8) the effect of switching from sync to hybrid is similar.

Why does this happen? Why do the arguments related to distributions spread in Section 6.2 not apply here? Our conjecture is as follows. Under the α mechanism, the much higher abundance of transition possibilities provided by the *hybrid* over the *sync* mode for system (P<α>${Q)}^{hybrid}$ causes a *reduction* of the distance between distributions $x_{coop}$ and $x_{indep}$ in $\phi^{hybrid}=d\left(x_{coop},x_{indep}\right)$, as discussed. On the contrary, in system ${\left(P|\beta |Q\right)}^{hybrid}$, written more accurately $P^{hybrid}\left|\beta \right|Q^{hybrid}$, the higher abundance of transitions in $P^{hybrid}$ and $Q^{hybrid}$, with respect to those in $P^{sync}$ and $Q^{sync}$, offers *more opportunities* to $P^{hybrid}\left|\beta \right|Q^{hybrid}$ than to $P^{sync}\left|\beta \right|Q^{sync}$ to perform *synchronization transitions* involving *P* and *Q* (the reader is invited to recall the discussed difference between *sync* execution mode of a bool net and *synchronization transition* for parallel composition *P*|β|*Q*). More synchronization transitions for $P^{hybrid}\left|\beta \right|Q^{hybrid}$ yield a *bigger gap* between distributions $post_{P|\beta |Q}^{hybrid}\left(X_{PQ}\right)$ and $post_{P|||Q}^{hybrid}\left(X_{PQ}\right)$ (P|||Q cannot perform any synchronization transition), or between distributions $pre_{P|\beta |Q}^{hybrid}\left(Y_{PQ}\right)$ and $pre_{P|||Q}^{hybrid}\left(Y_{PQ}\right)$ (switching from forward to backward reasoning), the latter being the distributions that feature in the definition of $\phi_{P|\beta |Q}^{m}\left(Y_{PQ}\right)$ in Equation (19).

The conclusion is that the comparison among α, β and γ cannot be done independently of the execution mode.

### 9.3. Mechanisms: α vs. β vs γ

We come finally to the comparison among cooperation mechanisms, one that we must contextualize to one or the other execution modes, as just established.

For further assessment of the data in Figure 9, we have included in the plots of Row 1, relative to the dkl distance, a gridline corresponding to the expected relative entropy $dkl[rand_{1}||rand_{2}]$, where $rand_{1}$ and $rand_{2}$ are two *random* distributions. Similarly, in the plots of Rows 2 and 3, relative to Manhattan distance, gridlines indicate the expected Manhattan distance $Manh[rand_{1}||rand_{2}]$. These expected values are established by the next two propositions.

**Proposition** **4.** *If $p=\{p_{1}\ldots p_{N}\}$ and $q=\{q_{1}\ldots q_{N}\}$ are random discrete distributions over the same domain of size N, where each element $p_{i}$ (respectively, $q_{i}$) is obtained by picking a real number $x_{i}$ (respectively, $y_{i}$) at random in (0, 1] and normalizing it so that p (respectively, q) is a probability vector, then as N→∞ the expected KL-divergence between p and q is:*

(32) Mean(dkl[p||q])=1/ln(4)=0.72.

**Proof.**  We have:

(33) $$\begin{array}{ccc} Mean(dkl[p||q]) & = & Mean(\sum_{i=1}^{N}\left(p_{i}Log_{2}\left(p_{i}/q_{i}\right)\right)) \end{array}$$

(34) $$\begin{array}{cc} = & Mean(\sum_{i=1}^{N}\left(\frac{x_{i}}{N/2}Log_{2}\left(x_{i}/y_{i}\right)\right)) \end{array}$$

(35) $$\begin{array}{cc} = & 2Mean(x_{i}Log_{2}\left(x_{i}/y_{i}\right)) \end{array}$$

(36) $$\begin{array}{cc} = & 2\int_{0}^{1}\int_{0}^{1}xLog_{2}\left(x/y\right)dxdy \end{array}$$

(37) $$\begin{array}{cc} = & 2/ln(16) \end{array}$$

(38) $$\begin{array}{cc} = & 1/ln(4). \end{array}$$

For Equation (34), we use the definitions of $p_{i}$ and $q_{i}$, and $Mean(\sum_{i=1}^{N}x_{i})=N/2$. For Equation (35), we swap *Mean* and *summation* and use the fact that $Mean(x_{i}Log_{2}\left(x_{i}/y_{i}\right))$ is the same for all *i*s. In Equation (36), we express the *Mean* as an integral on the unit square. The integral is routinely solved by parts, yielding Equations (37) and (38), where “*ln*” is the natural logarithm. □

**Proposition** **5.** *If p and q are random distributions of length N as defined in Proposition 4, then as N→∞ the expected Manhattan distance between p and q is:*

(39) Mean(Manh[p,q])=2/3.

**Proof.**  The easy proof is analogous to that of Proposition 4, and is omitted. □

#### 9.3.1. Comparing Mechanisms under the *sync* Mode

The relevant plots for this comparison are those in Column 1 of the grid in Figure 9, Row 2 or 3. $\bar{\phi }$ values for the α mechanism are remarkably higher than those attained by the β and γ mechanisms, which are very close to each other.

Bool nets executing in the *sync* mode exhibit a fully deterministic behavior, and, due to their simplicity, are probably the model most widely used in the literature for illustrating the basic concepts of IIT. If we were to accept them as a sufficiently realistic model for consciousness phenomena, then, according to our findings, we would conclude that the traditional, simple α cooperation mechanism, outperforms the alternative and, in a way, more sophisticated process-algebraic mechanisms β and γ in achieving high values of integrated information and, potentially, consciousness. This gap is particularly marked in the central segment of the range of coupling values, where *P* and *Q* show an even balance of cooperation and independence. However, the ever-lasting debate on determinism vs. nondeterminism in the natural sciences must invest also the neurosciences, and, although we cannot provide an accurate picture of the status of this discussion in this field, we believe that the assumption of a fully deterministic model for the brain appears too restrictive, if not naive. The *hybrid* mode may then be a better option. (Of course, the nondeterministic *hybrid* mode appears a better option *if* we restrict ourselves to the relatively small family of models investigated in the paper, but there may well be other nondeterministic models that lend themselves to interesting and perhaps more appropriate and realistic applications to neuroscience. For example, an option that seems to have gained attention inside the IIT community (as emerged in a private communication) is that of noisy mechanisms run in *sync* mode, e.g., the idea that the computations performed by the boolean functions associated with bool net nodes are affected by some percentage of error.)

#### 9.3.2. Comparing Mechanisms under the *Hybrid* Mode

The *hybrid* mode produces nondeterministic behavior. Nondeterminism *may* appear as a desirable feature, when dealing with complex systems and models in neuroscience; however, it is certainly a *must* for system models in Software Engineering. In the early phases of software development, for example, nondeterminism is typically used to prevent premature design choices that are postponed to later phases, down to the final implementation—a convenient way to offer implementation freedom. Then, if we set up to assess the three cooperation mechanisms in the context of formal models for Software Engineering, or system engineering in general, we believe that it is much more appropriate to refer to the hybrid execution mode.

Here, the relevant plots are those in the last column of Figure 9, Row 2 or 3. The $\bar{\phi }$ plots for the LOTOS-inspired mechanisms β and the CCS-inspired mechanism γ end up performing in a similar way, as it happens under the sync mode. However, the difference between α, on the one hand, and β-γ, on the other hand, is now considerably reduced, and a faster growth of the α plot in the lower part of the *coup* range is counterbalanced by the slightly higher values of the β-γ plots in the upper part.

It seems arduous, and perhaps even pointless, to speculate on the *detailed* differences among those three plots, trying to justify them formally—detailed differences that one may well expect, given the substantial difference between the *sharVar* and the *sharTrans* mechanisms. On the other hand, by taking a *coarser* look at the mentioned plots, we can reasonably conclude that, in terms of integrated information, *the performances of the three mechanisms are roughly equivalent.* Furthermore, by comparing their plots with the reference values (the gridlines) that derive from purely random distributions, we can additionally claim that all three methods of coupling two systems *P* and *Q* for them to interact, *do their jobs quite well*: in their highest values, all of them roughly double those reference values.

## 10. Conclusions

In this paper, we have addressed the scenario of two state transition systems *P* and *Q* that exhibit different types of cooperation and a variable degree of coupling. We have applied the informational measure of averaged *Integrated Information* $\bar{\phi }$ for the assessment and comparison of two fundamentally different cooperation mechanisms: (i) the standard *shared variables* mechanism associated to bool nets and very often adopted in the IIT literature, expressed as *P*<α>*Q*; and (ii) the *shared transitions* mechanism typical of process algebras, which we have studied in the two forms *P*|β|*Q* and P[γ]Q. In each case, α, β and γ control and measure the degree of coupling between *P* and *Q*. Having been able to export $\bar{\phi }$ from its standard application context and to adapt it to a completely novel field, re-defining what *cooperation* and *independence* mean in the new setting, is, in our opinion, one of the interesting and original contributions of our work, on the conceptual side.

We have modeled *P* and *Q* as boolean nets, and have considered three possible *execution modes*, namely *synchronous*, *asynchronous* and *hybrid*, although the anomalies of the second mode soon suggested to drop it. Furthermore, we have considered two variants of Integrated Information, based on two distinct measures of *distribution distance*, namely Kullback–Leibler divergence “*dkl*” and Manhattan distance, which avoids some limitations of dkl. With the main objective to compare the α, β and γ cooperation mechanisms (*coop*), the idea to articulate our experimental analysis along those two additional dimensions—execution modes (*m*) and distribution distances (*dd*)—has been useful for obtaining a sufficiently large set of plots for

$${\bar{\phi }}_{dd}^{m}\left(\text{P-}coop\text{-Q}\right)$$

on which to ponder.

In summary, the inspection of these plots has led us to the following main conclusions.

- Adopting a definition of $\bar{\phi }$ based on Manhattan distance rather than dkl makes averaged integrated information more widely applicable; furthermore, when both variants apply, they yield nicely compatible indications. For our purposes, Manhattan distance is therefore more convenient, and safe. It is worth noting that the Earth Mover’s Distance (EMD) [17], adopted in IIT 3.0 [2] but computationally more costly that Manhattan distance, would also avoid the mismatch problem arising with dkl.
- Under the deterministic, sync execution mode, the IIT-standard cooperation mechanism α performs considerably better than β and γ, especially for bipartite systems structured so that the two parts exhibit an intermediate degree of coupling. Conversely, under the nondeterministic hybrid mode, which may be more appropriate for cognitive system models, but is definitely more appropriate for Software Engineering models, the three mechanisms exhibit roughly equivalent performances, and *good* ones, at least compared with those achieved by using randomized state distributions.

Could the latter approximate equivalence be intuitively expected a priori? For the author, this was not the case. Let us explain.

In the general context of discrete state-transition models for distributed, concurrent systems, it seems reasonable to consider a cooperation mechanism as *effective* when the cooperating parts can produce state distributions—*successor* or *predecessor* state distributions, corresponding to *effect*- or *cause*-reasoning—that are markedly different from those achieved when the parts work independently. The reason one might expect, at least for the hybrid mode, higher $\bar{\phi }$ values for the *sharTrans* than for the *sharVar* mechanism has to do with the difference in *intrinsic complexity* of the two mechanisms.

For simplicity, let us refer to effect reasoning, i.e., successor state distributions. For finding the next state $y_{PQ}$ under α-cooperation, we only need to evaluate the *n* boolean functions of the bool net, where *n* is the overall number of nodes; the interactivity between *P* and *Q* comes “for free”, depending only on the fact that, for intermediate values of α, both *P* and *Q* read a mix of local and remote nodes.

Under β- and γ-cooperation, boolean function evaluation is still necessary, but then the possible local transition labels must be computed, and these depend both on current local states $x_{P}$ and $x_{Q}$ and on next states $y_{P}$ and $y_{Q}$, according to our labeling policy. Then, transition $x_{PQ}\to y_{PQ}$ is determined after a sort of negotiation between *P* and *Q*, based both on the locally available labeled transitions and on the set β of synchronization labels. It is clear that mechanisms β and γ are intrinsically more complex than α: they manipulate more information and do more work. It seemed then plausible that these mechanisms were able to exploit this additional information and machinery for creating next state distributions $y_{coop}$ that can depart more markedly from the reference distribution $y_{indep}$, thus achieving higher $\bar{\phi }$ values. (We also expected that the noise injected in P∗ and Q∗ for computing the distribution product $pre_{P*}^{m}\left(Y_{P}\right)\times pre_{Q*}^{m}\left(Y_{Q}\right)$ in Equation (15) could act as a limiting factor for the gap between this distribution and distribution $pre_{P<\alpha >Q}^{m}\left(Y_{PQ}\right)$, thus limiting the growth of ${\bar{\phi }}^{hybrid}\left(P<\alpha >Q\right)$, and keeping it well below ${\bar{\phi }}^{hybrid}\left(P|\beta |Q\right)$ and ${\bar{\phi }}^{hybrid}\left(P\left[\gamma \right]Q\right)$.)

Experimental evidence has shown that this expectation was wrong.

The combination of measure $\bar{\phi }$ with mechanisms β and γ may appear bizarre to the IIT expert (“why β and γ”) as well as to the expert in process algebra (“why $\bar{\phi }$”).

The first expert may criticize the adoption of additional interaction mechanisms for modeling brain-like systems, when several phenomena related to consciousness have already been successfully investigated by the plain bool net model without super-imposed features, and given that parallel compositions |β| and [γ] fail to satisfy conditional independence. Nevertheless, flat bool nets using the basic α mechanism suffer from serious *scalability* problems. The state space of an (n,k)-bool net has size $2^{n}$ and the associated tpm is a $2^{n}\times 2^{n}$ matrix: given this exponential growth, standard computers and algorithms can successfully deal only with “toy” models (such as those investigated in this paper), while there is no hope to handle realistic systems whose size *n* is, e.g., 5 or 10 orders of magnitude larger. We still believe that exploring macro-structured bool nets and higher-level interaction mechanisms—β, γ or others—may help alleviate those problems.

The process-algebra expert, in turn, may be puzzled by the fact that $\bar{\phi }$ is defined in terms of just *one-step* $x_{PQ}\to y_{PQ}$ of system behavior, but considers the full repertoire of conceivable system states, *including those unreachable from the initial state* (in this respect, $\bar{\phi }$, especially in the state-independent form, reflects the “counterfactual-reasoning” that informs J. Pearl’s Do-Calculus of intervention [9]) $\bar{\phi }$ may then appear: (i) inadequate to cope with systems in which the existence and importance of an initial state is out of question—as in Process Algebra and Software Engineering; and (ii) insensitive to phenomena that emerge only with longer transition sequences, e.g., attractors (the interested reader may look at the demonstration in [18], where attractors play a role for the analysis of *asymptotic* mutual information between boolean net components *P* and *Q*). With respect to this objection, we do agree that the two analytical approaches of *one-step-from-all-states* and *all-steps-from-one-state* may address and reveal different system properties, but, of course, we can take them as *complementary* techniques and explore their potential synergy. In any case, using $\bar{\phi }$ in the context of formal models and languages for software engineering is, to our knowledge, a radically novel way to assess the “power” of their structuring principles and operators.
